# Resolution Enhancement of Metabolomic J-Res NMR Spectra Using Deep Learning

**DOI:** 10.1021/acs.analchem.4c00563

**Published:** 2024-07-11

**Authors:** Yan Yan, Michael T. Judge, Toby Athersuch, Yuchen Xiang, Zhaolu Liu, Beatriz Jiménez, Timothy M. D. Ebbels

**Affiliations:** † Section of Bioinformatics, Division of Systems Medicine, Department of Metabolism, Digestion and Reproduction, Faculty of Medicine, Imperial College London, London W12 0NN, U.K.; ‡ National Phenome Centre, Department of Metabolism, Digestion and Reproduction, Imperial College London, London W12 0NN, U.K.; § Department of Mathematics, Imperial College London, London SW7 2AZ, U.K.

## Abstract

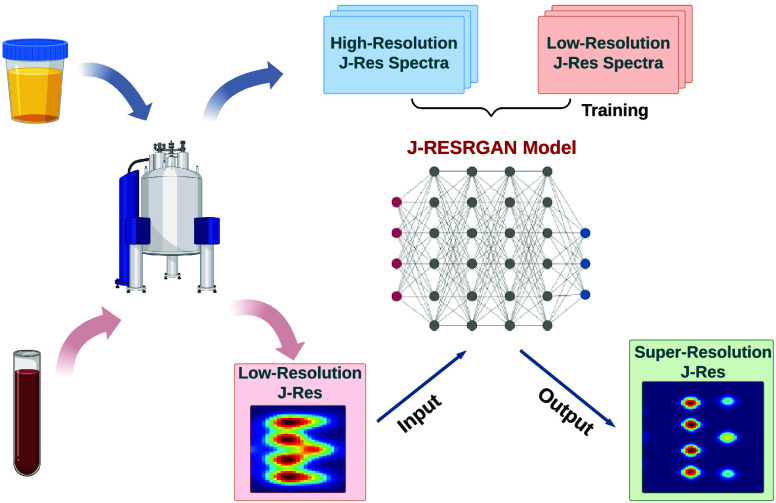

J-Resolved (J-Res) nuclear magnetic resonance (NMR) spectroscopy is pivotal in NMR-based metabolomics, but practitioners face a choice between time-consuming high-resolution (HR) experiments or shorter low-resolution (LR) experiments which exhibit significant peak overlap. Deep learning neural networks have been successfully used in many fields to enhance quality of natural images, especially with regard to resolution, and therefore offer the prospect of improving two-dimensional (2D) NMR data. Here, we introduce the J-RESRGAN, an adapted and modified generative adversarial network (GAN) for image super-resolution (SR), which we trained specifically for metabolomic J-Res spectra to enhance peak resolution. A novel symmetric loss function was introduced, exploiting the inherent vertical symmetry of J-Res NMR spectra. Model training used simulated high-resolution J-Res spectra of complex mixtures, with corresponding low-resolution spectra generated via blurring and down-sampling. Evaluation of peak pair resolvability on J-RESRGAN demonstrated remarkable improvement in resolution across a variety of samples. In simulated plasma data, 100% of peak pairs exhibited enhanced resolution in super-resolution spectra compared to their low-resolution counterparts. Similarly, enhanced resolution was observed in 80.8–100% of peak pairs in experimental plasma, 85.0–96.7% in urine, 94.4–98.9% in full fat milk, and 82.6–91.7% in orange juice. J-RESRGAN is not sample type, spectrometer or field strength dependent and improvements on previously acquired data can be seen in seconds on a standard desktop computer. We believe this demonstrates the promise of deep learning methods to enhance NMR metabolomic data, and in particular, the power of J-RESRGAN to elucidate overlapping peaks, advancing precision in a wide variety of NMR-based metabolomics studies. The model, J-RESRGAN, is openly accessible for download on GitHub at https://github.com/yanyan5420/J-RESRGAN.

Nuclear Magnetic Resonance (NMR) spectroscopy is an indispensable tool for elucidating molecular structure and dynamics in various scientific disciplines, especially metabolomics. This is in part due to its high reproducibility and robustness, and also its ability to analyze of a wide variety of sample types requiring little sample preparation. J-Resolved (J-Res) NMR is distinguished by its capability to separate scalar coupling information from the chemical shift, facilitating a deep and interpretable structural description of metabolites. In complex mixtures such as those analyzed in metabolomics, J-Res offers a practical complement to one-dimensional (1D) data due to its ability to disperse overlapping peaks from 1D spectra into a second dimension while being significantly faster to acquire than most other two-dimensional (2D) experiments. Yet analytical challenges persist, especially in the crowded regions of the spectrum where overlaps remain. These overlapping signals can hinder the precise assignment and quantification of individual resonances. While higher resolution can be attained at the expense of much higher acquisition times, deconvolution of J-Res NMR spectra remains both important and challenging as computational approaches are normally quicker and more cost-effective.

A variety of computational methods have been employed to deconvolute 2D NMR spectra for several decades. Among these, nonuniform sampling (NUS) is employed during the acquisition phase of NMR experiments, serving to enhance spectral resolution and resolve complex structures.^1^ Additionally, other traditional methods, including maximum entropy^2^ and linear prediction,^3^ have predominantly addressed signal overlaps within the processing of time-domain NMR data. However, some of these conventional approaches can only be applied at the time of experimental set up and others are of varying performance. More recently, some methods have been developed, shifting focus toward frequency-domain and processed NMR data to directly resolve the overlaps. Notably, Chylla et al. introduced a parametric spectral deconvolution algorithm termed fast maximum likelihood reconstruction (FMLR) tailored for accurate signal quantification in 2D ^1^H–^13^C NMR spectra.^4^ Additionally, Heinecke et al. developed a Bayesian algorithm based on BATMAN^5^ to perform automated metabolite deconvolution and quantification in 2D J-Res NMR spectra.^6^ Nevertheless, these methodologies still exhibit limitations. Some are not directly adaptable to J-Res NMR data, while others are constrained by their assumptions of peak shape and computational demands, rendering them unsuitable for handling a large collection of spectra.

Deep learning and artificial intelligence are becoming of increasing importance in many areas of science including NMR spectroscopy.^7^ In particular, deep learning offers a promising approach to addressing this deconvolution challenge. Li et al. introduced a deep neural network (DNN)-based approach called DEEP Picker for peak picking and deconvolution in both 1D and 2D NMR spectra;^8^ however, as it was designed for macromolecular spectra, it did not include 2D J-Res NMR spectra. The power of deep learning extends beyond this, particularly in the field of image super-resolution (SR).^9^ Super-resolution deep learning has been effectively applied in mass spectrometry imaging (MSI) and illustrated unparalleled ability in enhancing the resolution of MSI.^10^ Such success suggests the possibility that the technique might be applicable to resolution enhancement of complex NMR spectra.

We aim to fulfill this need by using super-resolution deep learning to enhance the resolution of metabolomic J-Res spectra. Real-ESRGAN, a generative adversarial network (GAN)-based super-resolution technique (more details in Supporting Information 1.1), has been demonstrated to be a very useful tool for enhancing resolution on various imaging data types.^11−13^ This study aims to adapt and finetune the Real-ESRGAN model specifically for J-Res NMR spectra, producing a model we term J-RESRGAN, in order to enhance spectral resolution. To do this, we introduced a novel symmetric loss function accounting for the vertical symmetry in J-Res NMR spectra. Additionally, to overcome the challenge of insufficient labeled training data in metabolomic data analysis, we employ simulation to generate synthetic J-Res spectra. We trained our model on simulated J-Res NMR spectra of complex mixtures and evaluated on both simulated and experimental J-Res data. The developed model significantly enhances J-Res resolution, offering improved data quality without increased acquisition time, thus improving information recovery in past and future NMR metabolomic studies.

## Methods

### Simulated Training Data

To generate a data set large enough to train the deep learning model, we simulated spectra of human urine. This Urine Training Set was built from pairs of high-resolution (HR) and low-resolution (LR) spectra. The HR spectra were simulated using linear combinations of experimental pure compound ^1^H 600 MHz J-Res spectra and contained mixtures of metabolites in varying quantities. Details of the metabolites, experimental data acquisition, and preprocessing of pure compounds and their associated concentrations are provided in Supporting Information 1.2 and 1.3. To maximize variety in the training data set, we simulated 5 mixture types using 6, 8, 15, 34, or 40 metabolites common in normal human urine as reported in HMDB version 5.0.^14^ The examples of simulated urine spectra can be found in Figure S1. Concentrations were sampled from a normal distribution with means and standard deviations listed in Table S1. Each of these mixture types was represented by 1000 spectra, culminating in the total data set size of 5000 spectra. Each HR J-Res spectrum had dimensions of 256 × 16,384 points corresponding to the F1 (J-coupling) axis from −39.1542 to 39.1542 Hz and the F2 (chemical shift) axis from −3.560 to 13.129 ppm, respectively.

**Figure 1:**
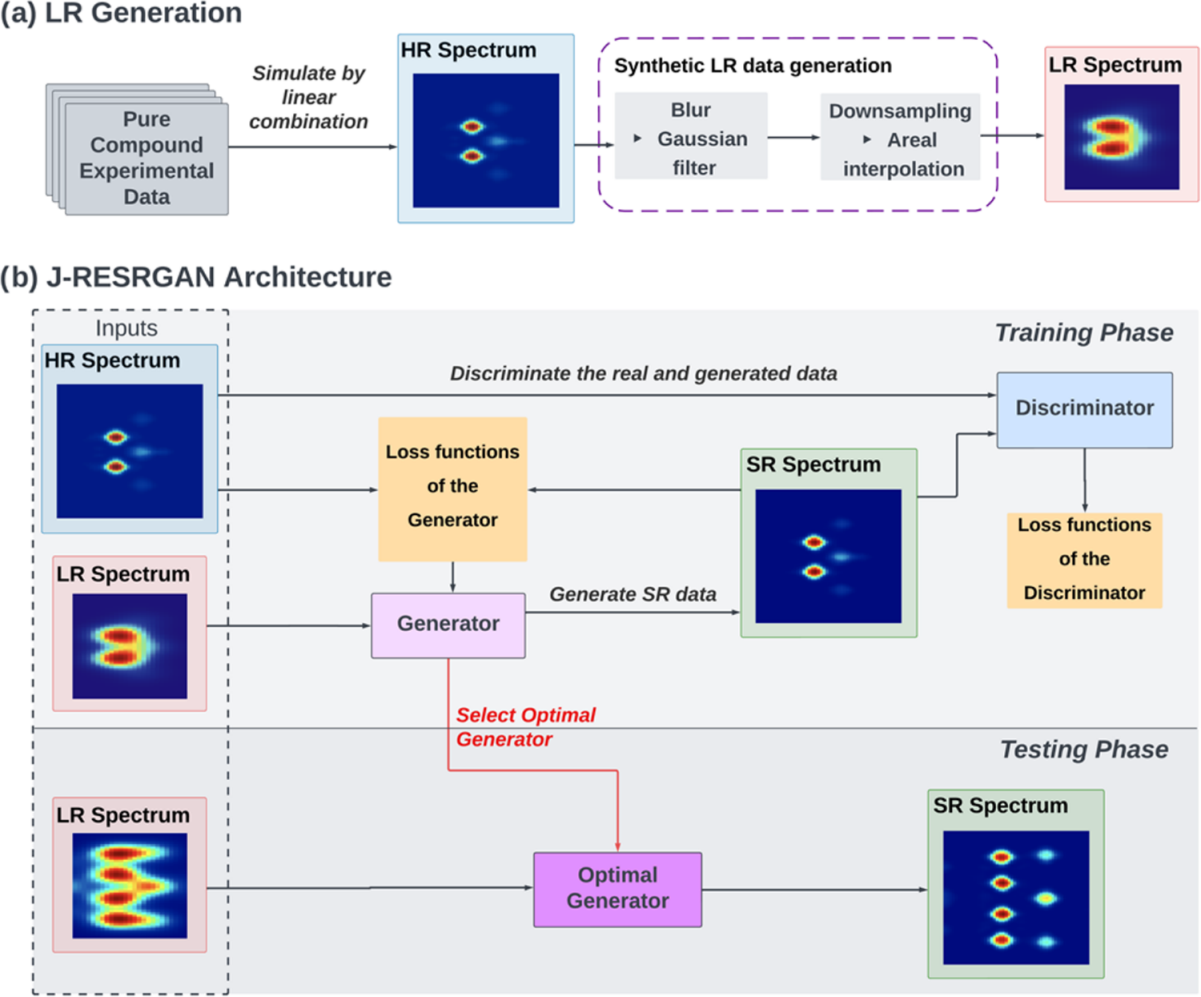
Workflow and architecture of **J-RESRGAN**. (a) Generation of high-resolution (HR) and low-resolution (LR) simulated data. (b) Architecture of J-RESRGAN showing how LR/HR pairs are used in training and testing phases.

For each HR spectrum, a corresponding LR spectrum was generated by applying a Gaussian filter with kernel dimensions (5, 7) to blur the HR spectrum, followed by down-sampling with areal interpolation, as illustrated in Figure 1a. As a result, the dimensions of each LR J-Res spectrum were reduced to 128 (F1) × 8192 (F2) pixels, effectively halving the resolution of the HR spectrum along both axes.

### Simulated Testing Data

We simulated HR and LR test spectra using the same approach used to generate the training data. However, to provide a more stringent test of the model, for test data, we simulated blood plasma mixtures (instead of urine) using a different set of metabolites (Table S2). Six unique mixtures were generated, corresponding to the top 1, 3, 6, 9, 12, and 15 metabolites in human blood plasma as reported by HMDB version 5.0.^14^ Each mixture type was represented by a single spectrum. The examples of simulated plasma spectra can be found in Figure S2.

### Experimental Testing Data

To establish the efficacy of the J-RESRGAN model, pairs of real HR and LR J-Res ^1^H NMR spectral data were analyzed. Four plasma pairs, 5 urine pairs, 2 milk pairs, and 2 juice pairs were acquired using a standard J-Res experiment as described in Supporting Information 1.5.^15^ In brief, several J-Res experiments of varying resolution were acquired by setting both the number of increments to 40, 80, and 160 and the number of data points in F2 to 8, 16, and 32K, denoted LR, HR, and HHR (higher HR), respectively.

We used these data to provide two challenges of resolution enhancement to the model. First, we tasked the model to enhance the LR data, comparing its output with HR. Second, as a more difficult test, we examined the effect of enhancing the HR data, comparing this to the HHR spectra. These different pairs were employed to substantiate the model’s robust performance across a variety of J-Res spectral data.

Moreover, the performance of J-RESRGAN was also evaluated against two traditional resolution enhancement techniques, nonuniform sampling and linear prediction, to further demonstrate the superiority of our model. The comparative analysis with NUS involved 3 examples of experimental J-Res spectra derived from one urine sample (see details in Supporting Information 1.6). The evaluation against linear prediction employed two experimental pairs of LR and HR spectra derived from plasma with different numbers of scans (see details in Supporting Information 1.7).

### Model Architecture and Training

J-RESRGAN was built upon the ×2 Real-ESRGAN^11^ which is a state-of-the-art model designed to perform super-resolution with a scale factor of ×2. The overall architecture of the modified J-RESRGAN model is depicted in Figure 1b. Within this structure, the generator^16^ is responsible for creating high-resolution, also called super-resolution (SR), spectra from low-resolution inputs, while the discriminator^17^ assesses the authenticity of these artificially generated spectra compared to real high-resolution data.

During the training phase, the data were randomly cropped into 256 × 256 subimages. Unlike the standard Real-ESRGAN model, several modifications were made for better compatibility with J-Res NMR spectra. First, we only selected the HR patches containing peaks, to avoid training on empty regions of the spectra. Second, while the standard Real-ESRGAN uses the “uint8” data type, our super-resolution spectral predictions employed “float32” due to the high dynamic range of the spectral data. Most importantly, Real-ESRGAN employs three foundational loss functions (Supporting Information 1.1): pixel loss for pixel-wise accuracy, perceptual loss^18^ for perceptual quality and feature similarity, and GAN loss^19,20^ to generate realistic images by discriminating between the real and generated data. To maximize performance, loss functions should exploit characteristics and symmetries of the data domain. A key feature of J-Res spectra is their symmetry about the *J* = 0 Hz line. In addition to the conventional loss functions, therefore, we introduced a novel symmetric loss function to account for this vertical symmetry.

The symmetry was assessed by comparing the upper and lower halves of each predicted SR spectrum using the Pearson correlation coefficient *r(x,y)*. To avoid the loss being dominated by the most intense peaks, we used the logarithm of the intensity data, and only consider columns of the image containing nonzero pixels.
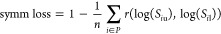
where *S_i_*_u_ and *S_i_*_l_ denote intensity vectors of the upper and lower half of the *i*th column, and the sum is taken over the *n* columns *P* containing nonzero pixels.

The J-RESRGAN model was trained on two NVIDIA GeForce GPUs (with each equipped with 4352 CUDA cores), each with a batch size of 1, leading to an aggregate total batch size of 2. We trained J-RESRGAN for 86K iterations with learning rate 1 × 10^–4^ for around 14 h (see Supporting Information 1.1 for more details). The remaining parameters adhered to the defaults of Real-ESRGAN. The optimal prediction model was selected based on the loss functions evaluated on independent simulated test data (Supporting Information 1.8).

### Performance Evaluation

A quantitative metric, we call the resolvability score, was devised to assess the efficacy of the model on peak deconvolution tasks. The motivation for this score is that the depth of the valley between two adjacent peaks serves to measure the extent their overlap. We formulate the score as
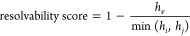
where *h*_*v*_, *h*_*i*_, and *h*_*j*_ symbolize the heights of the valley floor and the respective heights of two adjacent peaks (Figure S4). The score ranges between zero for unresolved peaks and one for a fully resolved peak pair.

To identify peak pairs, 2D peak detection was performed on each HR spectrum. Peak detection was conducted using the function *ng.analysis.peakpick.pick* of *nmrglue* package in Python. For every peak, the three nearest peaks were recorded, yielding 3 potential peak pairs. Peak pairs with distances surpassing 30 pixels (0.031 ppm or 18.6 Hz) were excluded. The HR positions of each peak pair were used in the corresponding LR and SR spectra to perform peak matching, thereby determining the resolvability scores.

The model, J-RESRGAN, tutorial and documentation are openly accessible for download on GitHub at https://github.com/yanyan5420/J-RESRGAN.

## Results and Discussion

We evaluated the ability of our J-RESRGAN model to enhance the resolution of metabolomic J-Res spectra using a combination of simulated and real data, focusing on the resolvability as a quantitative metric of performance. Using the pretrained network, enhancement of resolution can be obtained in a few seconds on a standard desktop machine.

### Validation on Simulated Data

#### Overview Analysis

**Figure 2:**
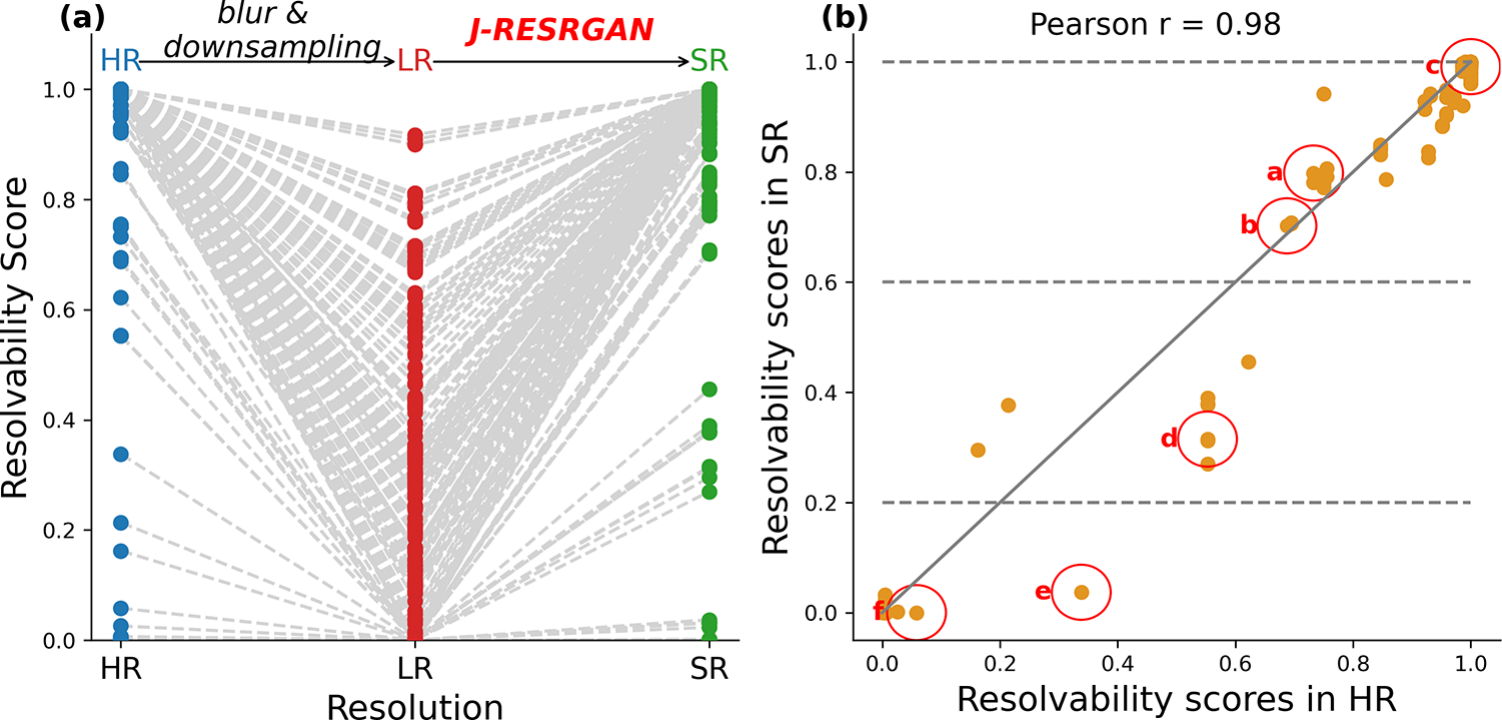
Plot (a) depicts the trend of resolvability scores transitioning from high-resolution (HR) spectra to low-resolution (LR) spectra and then to super-resolution (SR) spectra; each distinct line represents the trend for a single peak pair. Plot (b) illustrates the relationship of resolvability scores between HR and SR spectra. Highlighted peak pairs are shown in more detail in Figure 3. The gray line represents recovery of the original HR resolvability.

The performance of the proposed J-RESRGAN model, trained on simulated urine spectra, was assessed using six simulated plasma spectra. As depicted in Figure 2a, the trend of resolvability scores for 300 peak pairs across all six spectra predominantly adheres to a distinctive “*V*” shape. The descent observed on the left side illustrates the diminished resolution in the LR spectra, a consequence of both blurring and down-sampling. Conversely, the subsequent ascent on the right side, where 100.0% of peak pairs exhibit improved resolution in the SR, demonstrates the capability of the J-RESRGAN model to recover obscured detail, efficiently reconstructing the HR spectra from the degraded LR spectra.

Figure 2b compares the resolvability scores of HR and SR spectra for each peak pair. The correlation coefficient of 0.98 reveals a substantial agreement between the predicted SR and the ground truth HR resolvability scores, showcasing the model’s predictive ability. Notably, points positioned above the reference line indicate that the J-RESRGAN model not only recovers the original resolvability from poorly resolved LR spectra but occasionally surpasses the resolvability of the ground truth. Nonetheless, there are still a small number of pairs that could not be recovered to their original resolvability level; these tended to have low resolvability in the HR.

To facilitate interpretation, we categorize the resolvability scores into three distinct groups: 0–0.2 as poorly resolved, 0.2–0.6 as partially resolved, and 0.6–1.0 as well resolved. Peak pairs circled in red are representative of their respective categories. We investigated each of these examples to better understand the model’s performance.

#### J-RESRGAN Enhances Resolution of J-Res Spectra

**Figure 3:**
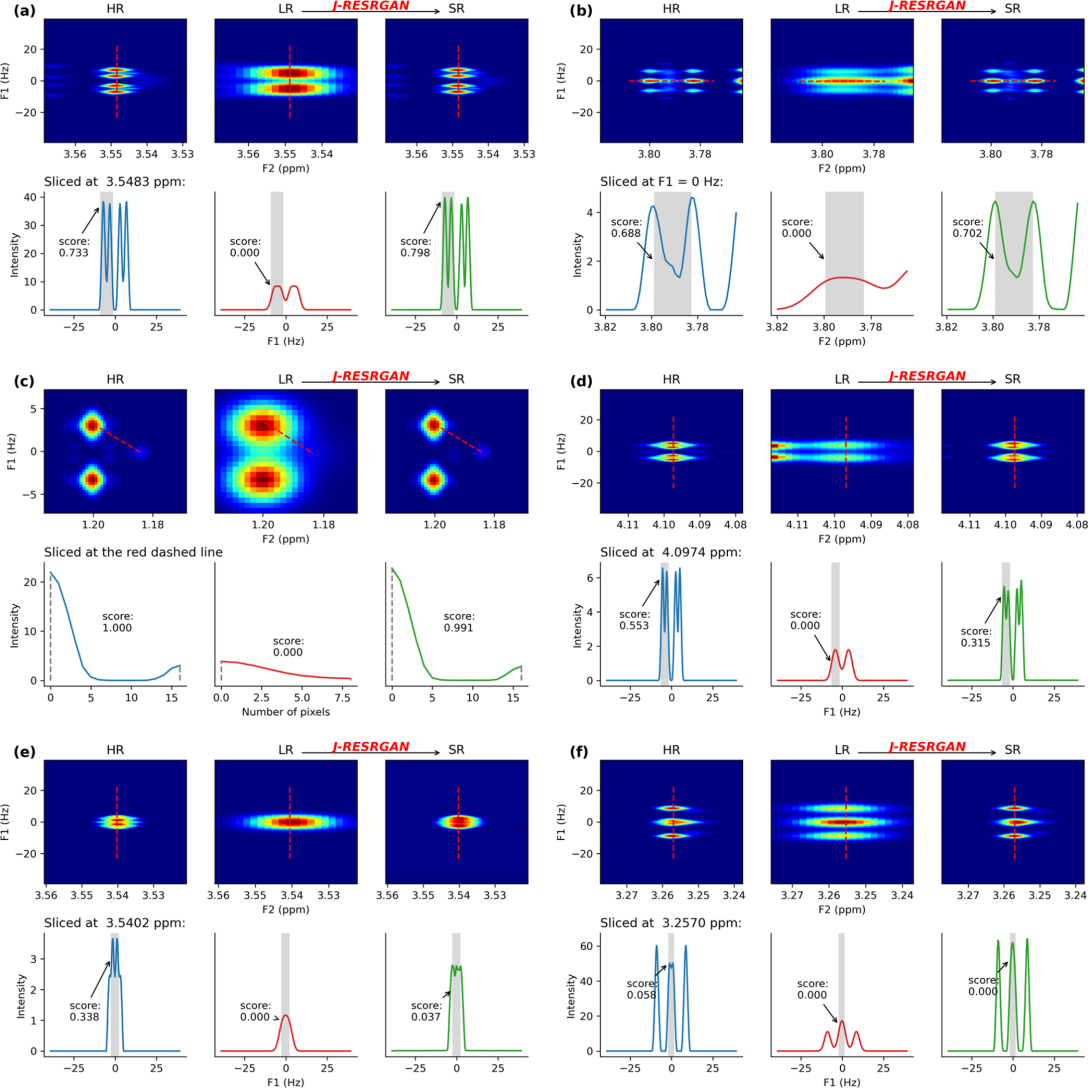
Examples of individual peak pairs from simulated test data. Panels (a–f) align with the peak pairs identified in Figure 2b by their respective labels. For each panel, the upper row displays the 2D HR, LR, and SR spectra, respectively; the lower row depicts the associated 1D profiles along the red dashed line; blue indicating HR, red indicating LR, and green indicating SR. Plot (b) shows a peak pair sliced along the F2 axis, and plot (c) shows the peak pair sliced along a diagonal axis; remaining plots show peak pairs sliced along the F1 axis.

The evaluation of six representative peak pairs is illustrated in Figure 3. Examples a, b, and c show three distinct peak pairs well resolved in HR. Each pair is aligned either along the F1 axis (Figure 3a), the F2 axis (Figure 3b), or positioned diagonally (Figure 3c). The transformation from a doublet in the LR to a doublet of doublets is notably evident in Figure 3a, while Figure 3b shows how the weaker signal in between the doublet is lost in the LR spectrum while it is recovered in SR. In Figure 3c, a prominent peak overlaps and obscures a smaller peak in the LR. J-RESRGAN successfully restored both peaks and effectively separates them. Such findings substantiate the model’s proficiency in enhancing the spectral resolution in a variety of orientations.

Figure 3d illustrates a partially resolved peak pair. The original coupling pattern—a doublet of doublets—is recovered in the SR, albeit with a slightly lower resolvability score than in the HR. Figure 3e,f shows examples of peaks which are poorly resolved in the HR. These become completely unresolved in the LR, and, consequently, are poorly resolved in the predicted SR. These results emphasize that the intrinsic resolvability of the HR serves as a pivotal determinant in the ability of the model to fully resolve peaks.

As expected, the efficacy and accuracy of J-RESRGAN is limited in particularly low-information cases. Peaks which had low intensity and low resolvability in the HR exhibited more errors (Figure 3d,e). However, there were also peaks with a similarly small averaged peak intensity for which excellent resolution was achieved in the predicted SR (Figure 3b), possibly due to a wider interpeak spacing as compared to Figure 3d,e. This observation suggests that, as factors involved in resolvability, both peak intensity and distances between members of a peak pair influence the model’s performance. This knowledge may be useful in predicting the accuracy for reconstruction of a given peak.

#### Peak Distance is the Dominant Factor in J-RESRGAN’s Resolution Enhancement

Contrary to the initial expectation, the averaged peak intensity shows negligible correlation with the model performance, as detailed in Figure S5. Therefore, we explored the influence exerted by the distance between members of each peak pair. Since peaks may be separated in the F1, F2, or both axes (diagonal separation), for consistency, we report peak distances in pixels.

**Figure 4:**
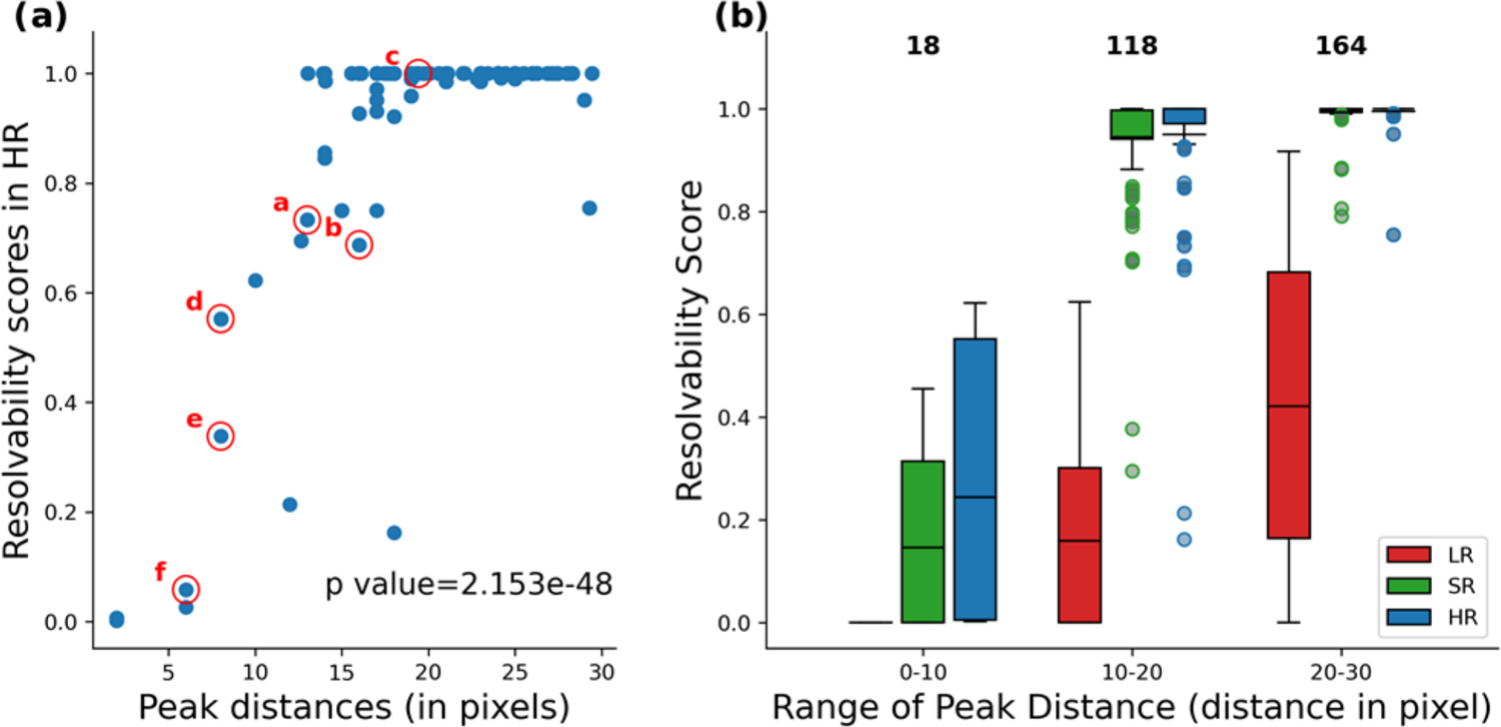
Plot (a) depicts the relationship between peak distances and resolvability scores in HR; highlighted peak pairs correspond to the examples in Figure 3. Plot (b) is the distribution of resolvability scores for varying peak distances, spanning all 300 peak pairs. In general, blue indicates HR, red for LR, and green for SR.

Figure 4a reveals a significant relationship between peak distance and resolvability scores in the HR. Owing to the strong correlation between the resolvability of HR and SR (Figure 2b), we can infer that peak distance also affects the resolvability scores in the SR. This affirms the influence of the peak distance on the model’s efficacy.

To explore peak distances further, we stratified them into five distinct ranges, each spanning 10 pixels (around 0.01 ppm or 6 Hz), as illustrated in Figure 4b. It is evident that the resolvability in the LR improves as peaks become further apart; however, it remains consistently inferior to both the HR and SR across all distance ranges. In the comparison between HR and SR spectra, it becomes apparent that the resolvability of SR mirrors that of the HR closely, with slight deviations only in scenarios with minimal peak distances.

This analysis of peak distances provides an explanation for the disparate resolvability observed in Figure 3b,e: 16 pixels in 3b versus 8 in 3e, despite similar peak intensities. Similarly, despite similar peak patterns, the resolution difference between Figure 3a,d is due to a larger peak distance in 3a (13 pixels) compared to 8 pixels in 3d.

### Validation on Experimental Data

To substantiate the above findings, further validation using experimental data is necessary. Examples of experimental urine and plasma J-Res spectra are given in Figures S6 and S7. In this subsection, we present results from one pair of LR and HR data for one plasma sample as an exemplar.

#### Overview Analysis

Utilizing the LR spectrum as an input, the J-RESRGAN model was employed to generate the predicted SR spectrum. In the experimental data, the observed peak separations exceed those in the simulated data; consequently, peak pairs with distances surpassing 25 pixels (around 0.025 ppm) have been excluded. 68 peak pairs meeting the max distance criterion were found in the HR spectrum. Small shifts were observed between the peaks in the SR and HR spectra; thus, we devised an algorithm (Supporting Information 2.3) to precisely match peak pairs at the two resolutions.

**Figure 5:**
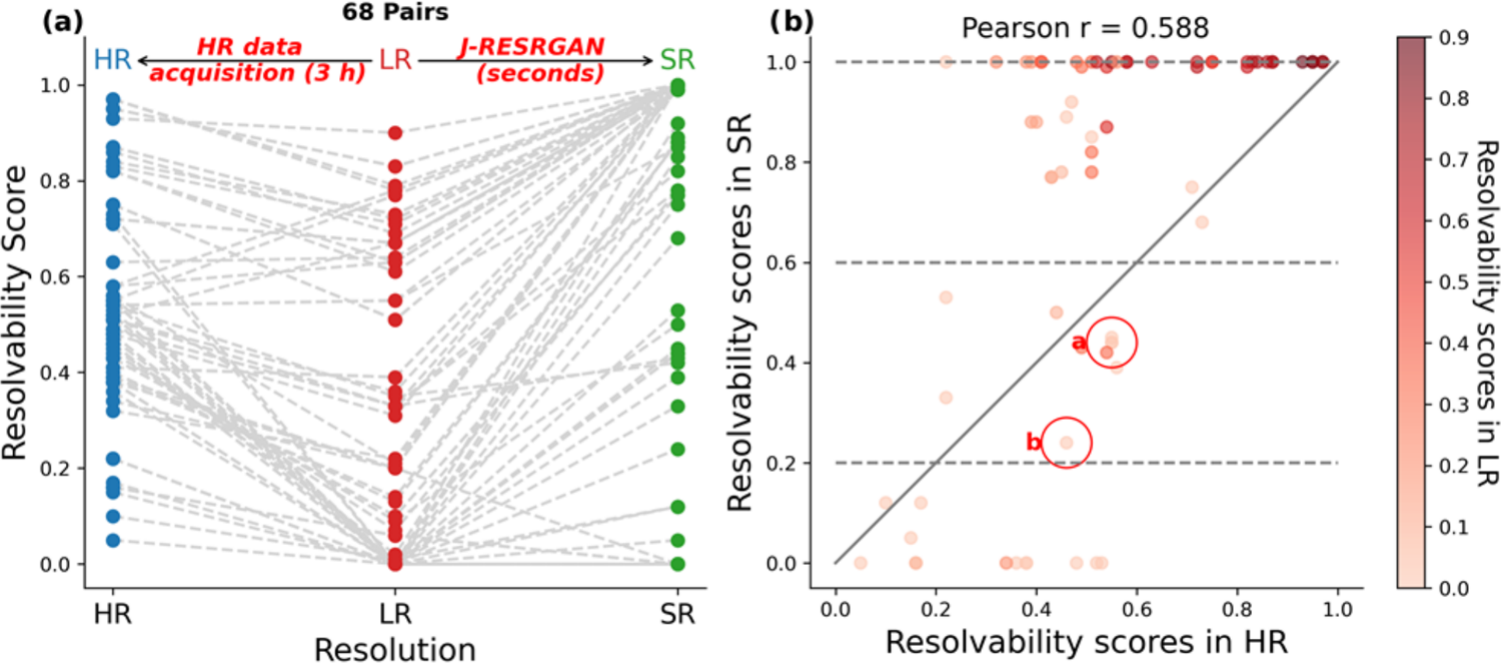
Resolvability scores for matched peak pairs. (a) Trend from LR to experimental HR (longer J-Res acquisition) and to SR predicted by J-RESRGAN. (b) Comparison for each peak pair between HR and SR. Points are colored by the resolvability scores in LR. Highlighted peak pairs are shown in more detail in Figure 6.

Figure 5a delineates the overall trends of resolvability scores across HR, LR, and SR spectra for all peak pairs, again exhibiting a prominent “*V*” shape. Notably, 65/68 (95.6%) of these peak pairs demonstrate enhanced or equivalent resolution in the SR spectra compared to the LR, indicating an overall improvement on the resolvability of the predicted SR. As shown in Figure 5b, a high proportion of peak pairs demonstrate enhanced resolvability in the SR compared to the HR, as indicated by the data points above the reference line. This implies that the resolvability of the predicted SR can be commensurate with, or even exceed, that of the HR.

Obtaining high-resolution J-Res data experimentally is a process typically spanning several hours. In contrast, the J-RESRGAN model only requires few seconds to produce a super-resolution spectrum from low-resolution input on a standard laptop, underscoring the practical utility of the approach.

A small subset of peak pairs did not demonstrate enhanced resolution, which can be attributed to their inherently low resolvability in both LR and HR spectra.

#### Detailed Analysis

**Figure 6:**
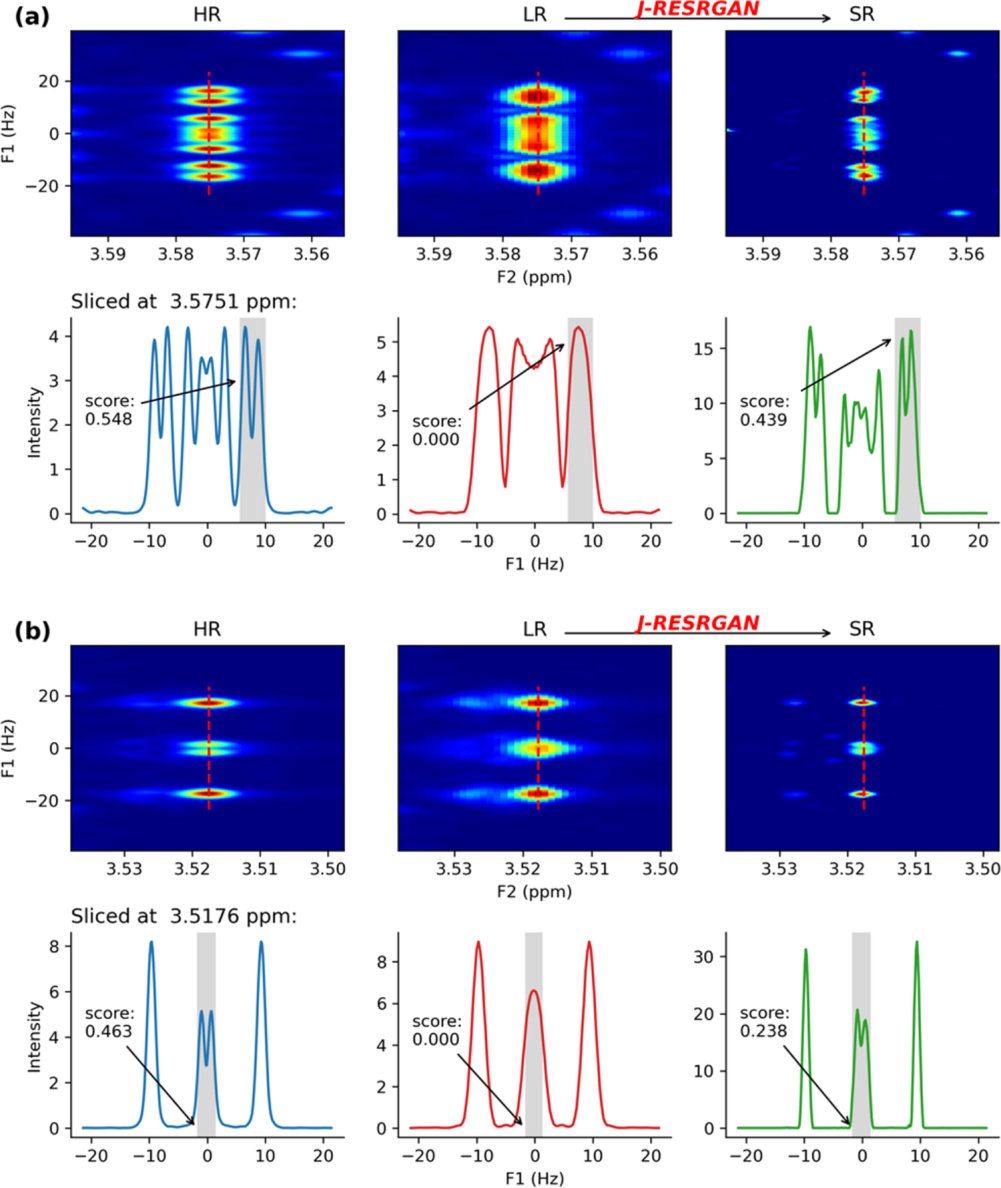
Examples of peak pairs from experimental data. For each individual panel, the upper row displays the 2D HR, LR, and SR spectra respectively; the subsequent row depicts the 1D profiles along the red dashed line; blue—HR, red—LR, and green—SR. Plots (a, b) display two examples of peak pairs aligned along the F1 axis, demonstrating the recovery to doublets in the SR spectra from the singlets observed in LR spectra.

Figure 6 provides detailed information for several peak pairs found in the experimental data. Figure 6a,b showcases examples with enhanced resolution. Despite only a single peak appearing in the LR (shaded region), the model correctly recovers two peaks in the SR. While the SR is not perfectly symmetrical, it mirrors a coupling pattern akin to the HR, underscoring the model’s capability in peak deconvolution.

The peak pair in Figure 6a exhibits a superior resolvability score in the SR compared to that in Figure 6b. The difference can be explained by the larger different interpeak distance for the former (15 pixels) as compared to the latter (11 pixels). In agreement with the simulated data, this underscores the importance of peak distance as a pivotal factor in model performance.

All other examples, including other plasma (Figure S8), urine (Figures S9 and S10), full fat milk (Figure S11), and orange juice (Figure S12), give similarly good results, with enhanced resolution observed in 80.8–100% of experimental plasma peak pairs, 85.0–96.7% in urine, 94.4–98.9% in full fat milk, and 82.6–91.7% in orange juice.

In summary, the J-RESRGAN model demonstrates a pronounced aptitude for enhancing resolvability within experimental J-Res metabolomic spectra (Figure 6a,b), albeit accompanied by some uncertainties, such as the suppression of very small peaks (Figure S13) and instances of over-resolution (Figure S14). These uncertainties highlight the importance of understanding the reliability of the predicted peaks.

#### Reliability Analysis

In this analysis, the HHR spectrum, acquired with 160 increments, was taken to represent the ground truth for the existence and position of real peaks. We then compared this to the LR spectrum acquired with 40 increments, and SR spectrum generated by J-RESRGAN. A detection threshold of 0.5% of the maximum intensity was employed in the HHR to ensure comprehensive identification of ground truth peaks. Subsequently, varying thresholds [1, 5, 10%] were applied to detect peaks in the LR and SR spectra. Peaks from LR or SR were matched to those in the HHR data with a 2-pixel distance tolerance.

**Figure 7:**
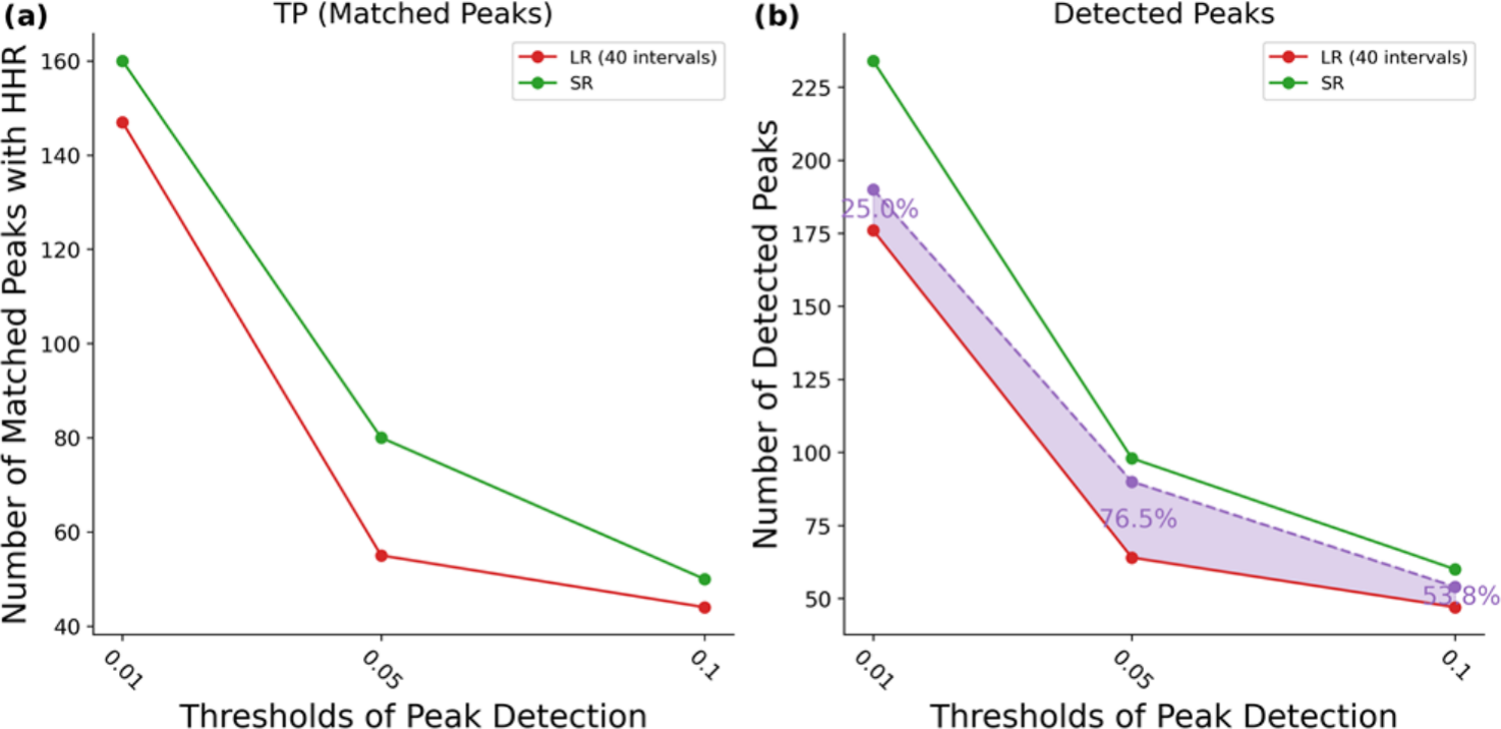
Peak reliability across detection thresholds. (a) The number of peaks matching those in the highest resolution data (HHR). (b) Number of detected peaks in LR and SR. Red—LR, green—SR. SR is the predicted J-RESRGAN output with LR as input. The purple region shows the fraction of gained information that is reliable.

Figure 7a shows the number of peaks in LR and SR which match those found in the ground truth HHR. It is clear that the SR spectrum reveals a higher number of peaks that match with HHR (i.e., a higher number of true positives) at all thresholds compared to LR. Figure 7b shows the total number of peaks detected, where it is clear that the SR exhibits a greater number of peaks than LR. The purple region in Figure 7b indicates the proportion of these additional peaks in SR that are matched with those in HHR. When applying peak detection thresholds of 0.05 or 0.1, it is clear that the majority of the additional peaks in SR match peaks identified in HHR (76.5 and 53.8% respectively). This observation attests to the reliability of the J-RESRGAN model in generating true peaks. However, when looking at smaller peaks (threshold of 0.01), only 25.0% of the extra peaks in SR correspond with those in HHR, implying that low-intensity peaks may either represent artifacts or over-resolved peaks (Figure S14) requiring verification against an even higher-resolution J-Res spectrum.

As expected, SR spectra generated by the J-RESRGAN model exhibit more dependable peaks at higher intensities. Nevertheless, the absence of corresponding peaks in HHR does not invalidate predicted peaks, as they may be over-resolved peaks verifiable by higher-resolution spectra. Our current methodology does not allow determination of specific reliability scores to individual peaks. Future work should focus on the development of a model capable of quantifying confidence levels for each predicted peak, thereby enhancing the reliability of the super-resolved spectra.

#### Comparison to Conventional Methods

In this comparative analysis, J-RESRGAN was evaluated against experiments with double the resolution and hence longer experimental times, as well as two conventional resolution enhancement techniques: NUS, which operates at the acquisition stage, and linear prediction, which works during the processing stage of time-domain NMR data. J-RESRGAN, working on postprocessed frequency-domain NMR data, diverges in its approach from these traditional methods. Despite this, it consistently shows statistically significant enhancement over both NUS (Supporting Information 2.10) and linear prediction (Supporting Information 2.11). Remarkably, J-RESRGAN’s superior performance is not limited by the number of scans during spectral acquisition, indicating its robust functionality even when applied to spectra acquired with fewer scans (Figures S15–S17 and S19). Crucially, J-RESRGAN not only demonstrates an exceptional ability to enhance resolution in spectra obtained through standard acquisition protocols but also shows efficacy with spectra acquired via NUS (Figure S17). This underscores the model’s utility and efficiency across a range of acquisition techniques.

## Conclusions

In this study, we introduced a super-resolution enhancement model, named J-RESRGAN, equipped with a novel symmetric loss function to facilitate peak deconvolution on 2D J-Res NMR spectra. The model with this symmetric loss function indeed not only elevates the resolvability of peak pairs (Figure S20) but also enhances the symmetry in the predicted SR spectra (Figure S21).

By using J-RESRGAN, the majority of peak pairs can be precisely recovered into their original resolvability states quickly, typically in seconds, as reflected in the ground truth high-resolution spectra. The model provides good results for a wide variety of matrices, independently of the one used for training. As expected, the spatial distance between the peaks within a given peak pair markedly influences the model performance on peak separation. Notably, while the peak intensity does not affect the efficacy of peak separation, it does affect the reliability for individual peaks.

Despite the inherent constraints associated with a dependence on peak distances and the peak intensity in the ground truth spectra, this study provides new insights into the topic of NMR peak deconvolution. It highlights the potential of super-resolution techniques in the analysis of other NMR spectral data, including other 1D and 2D NMR experiments. Ultimately it is hoped that tools such as J-RESRGAN will aid NMR spectroscopists and metabolomics practitioners in generating high-resolution data in a shorter time than currently possible, thus enabling further advances in biological and medical science. It may also increase the range of applications for low-resolution instruments such as room temperature magnets, making this NMR technology accessible to a broader range of researchers around the world.

## Supporting Information Available

Details about model settings, data acquisition, examples of simulated and experimental J-Res NMR spectra for complex mixtures, and additional validation results on various samples, including plasma, urine, full fat milk, and orange juice (PDF)

## References

[ref1] Le Guennec A.; Dumez J. N.; Giraudeau P.; Caldarelli S. Resolution-enhanced 2D NMR of complex mixtures by non-uniform sampling. Magn. Reson. Chem. 2015, 53 (11), 913–920. 10.1002/mrc.4258. 26053155

[ref2] Hore P. J. NMR data processing using the maximum entropy method. J. Magn. Reson. (1969) 1985, 62 (3), 561–567. 10.1016/0022-2364(85)90229-X.

[ref3] Led J. J.; Gesmar H. Application of the linear prediction method to NMR spectroscopy. Chem. Rev. 1991, 91 (7), 1413–1426. 10.1021/cr00007a007.

[ref4] Chylla R. A.; Hu K.; Ellinger J. J.; Markley J. L. Deconvolution of two-dimensional NMR spectra by fast maximum likelihood reconstruction: application to quantitative metabolomics. Anal. Chem. 2011, 83 (12), 4871–4880. 10.1021/ac200536b. 21526800 PMC3114465

[ref5] Hao J.; Astle W.; De Iorio M.; Ebbels T. M. BATMAN—an R package for the automated quantification of metabolites from nuclear magnetic resonance spectra using a Bayesian model. Bioinformatics 2012, 28 (15), 2088–2090. 10.1093/bioinformatics/bts308. 22635605

[ref6] Heinecke A.; Ye L.; De Iorio M.; Ebbels T. Bayesian deconvolution and quantification of metabolites from J-resolved nmr spectroscopy. Bayesian Anal. 2021, 16 (2), 425–458. 10.1214/20-BA1208.

[ref7] Karunanithy G.; Shukla V. K.; Hansen D. F. Solution State Methyl NMR Spectroscopy of Large Non-Deuterated Proteins Enabled by Deep Neural Networks bioRxiv 2023 10.1101/2023.09.15.557823. 10.1038/s41467-024-49378-8 PMC11176362 38871714

[ref8] Li D. W.; Hansen A. L.; Yuan C.; Bruschweiler-Li L.; Brüschweiler R. DEEP picker is a deep neural network for accurate deconvolution of complex two-dimensional NMR spectra. Nat. Commun. 2021, 12 (1), 5229 10.1038/s41467-021-25496-5. 34471142 PMC8410766

[ref9] Chen H.; He X.; Qing L.; Wu Y.; Ren C.; Sheriff R. E.; Zhu C. Real-world single image super-resolution: A brief review. Inf. Fusion 2022, 79, 124–145. 10.1016/j.inffus.2021.09.005.

[ref10] Xiang Y.; Metodiev M.; Wang M.; Cao B.; Bunch J.; Takats Z. Enhancement of Ambient Mass Spectrometry Imaging Data by Image Restoration. Metabolites 2023, 13 (5), 669 10.3390/metabo13050669. 37233710 PMC10222327

[ref11] Wang X.; Xie L.; Dong C.; Shan Y. Real-Esrgan: Training Real-World Blind Super-Resolution with Pure Synthetic Data. In Proceedings of the IEEE/CVF International Conference on Computer Vision, 2021; pp 1905–1914.

[ref12] Kong J.; Ryu Y.; Jeong S.; Zhong Z.; Choi W.; Kim J.; Houborg R.; et al. Super resolution of historic Landsat imagery using a dual generative adversarial network (GAN) model with CubeSat constellation imagery for spatially enhanced long-term vegetation monitoring. ISPRS J. Photogramm. Remote Sens. 2023, 200, 1–23. 10.1016/j.isprsjprs.2023.04.013.

[ref13] Qin Y.; Hu J.; Han J. A2OURSR: Adaptive adjustment based real MRI super-resolution via opinion-unaware measurements. Comput. Med. Imaging Graph. 2023, 107, 102247 10.1016/j.compmedimag.2023.102247. 37224741

[ref14] Wishart D. S.; Guo A.; Oler E.; Wang F.; Anjum A.; Peters H.; Gautam V.; et al. HMDB 5.0: the human metabolome database for 2022. Nucleic Acids Res. 2022, 50 (D1), D622–D631. 10.1093/nar/gkab1062. 34986597 PMC8728138

[ref15] Dona A. C.; Jiménez B.; Schäfer H.; Humpfer E.; Spraul M.; Lewis M. R.; Nicholson J. K.; et al. Precision high-throughput proton NMR spectroscopy of human urine, serum, and plasma for large-scale metabolic phenotyping. Anal. Chem. 2014, 86 (19), 9887–9894. 10.1021/ac5025039. 25180432

[ref16] Wang X.; Yu K.; Wu S.; Gu J.; Liu Y.; Dong C.; Change Loy C. ESRGAN: Enhanced Super-Resolution Generative Adversarial Networks. Proc. Eur. Conf. Comput. Vis. 2018, 63–79.

[ref17] Schonfeld E.; Schiele B.; Khoreva A. A U-Net Based Discriminator for Generative Adversarial Networks. In Proceedings of the IEEE/CVF Conference on Computer Vision and Pattern Recognition, 2020; pp 8207–8216.

[ref18] Johnson J.; Alahi A.; Fei-Fei L. Perceptual Losses for Real-Time Style Transfer and Super-Resolution. In Computer Vision–ECCV 2016: 14th European Conference, Amsterdam, The Netherlands, October 11–14, 2016, Proceedings, Part II 14 Springer International Publishing, 2016; pp 694–711.

[ref19] Goodfellow I.; Pouget-Abadie J.; Mirza M.; Xu B.; Warde-Farley D.; Ozair S.; Bengio Y. Generative Adversarial Nets. In Advances in Neural Information Processing Systems, 27, 2014.

[ref20] Ledig C.; Theis L.; Huszár F.; Caballero J.; Cunningham A.; Acosta A.; Shi W. Photo-Realistic Single Image Super-Resolution Using a Generative Adversarial Network. In Proceedings of the IEEE Conference on Computer Vision and Pattern Recognition, 2017; pp 4681–4690.

