# Supplementary material for: Resolution Enhancement of Metabolomic J-Res NMR Spectra Using Deep Learning
Source: Anal Chem. 2024 Jul 11;96(29):11707–15. doi: 10.1021/acs.analchem.4c00563 (PMC11270528; doi:10.1021/acs.analchem.4c00563)
Supplement: Supplementary file 1 — ac4c00563_si_002.pdf [file ac4c00563_si_002.pdf]

Supporting Information

Resolution Enhancement of Metabolomic J-Res NMR Spectra Using Deep Learning

Yan Yan[1], Michael T. Judge[1], Toby Athersuch[1]†, Yuchen Xiang[1], Zhaolu Liu[3], Beatriz Jiménez[1,2], and Timothy M. D. Ebbels[1*]

[1]Section of Bioinformatics, Division of Systems Medicine, Department of Metabolism, Digestion and Reproduction, Faculty of Medicine, Imperial College London, London, W12 0NN, UK.

[2]National Phenome Centre, Department of Metabolism, Digestion and Reproduction, Imperial College London, London, W12 0NN, UK.

[3]Department of Mathematics, Imperial College London, London, SW7 2AZ, UK.

†Drug Metabolism and Pharmacokinetics, Oncology Research and Development, AstraZeneca, Cambridge, CB2 0AA, United Kingdom

Table of Contents

1. Methods *3*

1.1 Real-ESRGAN Settings 3

1.2 Standards Database: NMR Data Acquisition 3

1.3 Pre-processing Spectra for Standard Pure Compound and Experimental Mixtures 6

1.4 Simulated Mixture Spectra 6

1.5 Experimental J-Res Data of Complex Mixtures (plasma, urine, milk, orange juice) 9

1.6 Experimental Pairs of Spectra for Comparison with NUS 10

1.7 Experimental Pairs of Spectra for Comparison with Linear Prediction 11

1.8 Selection of Optimal J-RESRGAN Model 12

1.9 Resolvability Score Plot 13

2. Results *13*

2.1 Peak Intensity vs. Resolvability 13

2.2 Experimental Mixture Spectra 14

2.3 Matching Peaks between HR and SR Spectra 15

2.4 V Plot for Plasma Samples at 600 MHz 16

2.5 V Plot for Urine Samples at 600 MHz 16

2.6 V Plot for Urine Samples at 800 MHz 17

2.7 V plot for Milk at 600 MHz 18

2.8 V Plot for Orange Juice at 600 MHz 19

2.9 Examples of Poorly Resolved & Over Resolved Peak Pairs 20

2.10 Comparison between J-RESRGAN and NUS 21

2.11 Comparison between J-RESRGAN and linear prediction 25

2.12 Comparison between Models with & without Symmetric Loss Function 27

1. Methods

1.1 Real-ESRGAN Settings

Real-Enhanced Super Resolution Generative Adversarial Network (Real-ESRGAN), a practical image restoration application, is exclusively trained on synthetic data, enabling it to adeptly restore most real-world images. As with all GAN architectures, Real-ESRGAN is fundamentally structured on two primary models: the generator and the discriminator. The generator uses low-resolution images as input and produces corresponding high-resolution predictions, while the discriminator attempts to distinguish between the ground truth and predicted high-resolution data. These models engage in a competitive interaction, each striving to outperform the other, thereby fostering a continuous improvement in the quality of the images produced. Specifically, Real-ESRGAN is designed for natural image super-resolution tasks and video restoration.

In Real-ESRGAN, three traditional loss functions are used: pixel loss, perceptual loss, and GAN loss, with weights {1, 1, 0.1} respectively. Pixel loss measures the pixel-wise accuracy between the generated image and the ground truth image. Here, pixel loss is implemented using L1 loss, also known as Mean Absolute Error (MAE), which ensures that the generated image retains structural integrity and details that closely match the ground truth image. Perceptual loss assesses the perceptual quality and feature similarity between the generated and ground truth images. Real-ESRGAN works by comparing high-level features extracted from a pre-trained deep neural network, called VGG19, to capture the perceptual differences rather than just pixel-wise differences. This loss function ensures the generated image not only looks similar pixel-wise but also feels similar in terms of textures and patterns to the human eye. GAN loss is derived from the method of Generative Adversarial Networks (GANs), where two networks, a generator and a discriminator, are trained and competing simultaneously. The generator aims to generate images that are indistinguishable from the ground truth, while the discriminator aims to differentiate between the predicted and real images. In Real-ESRGAN, GAN loss helps generate high-quality and realistic images, as the generator learns how to produce images to effectively fool the discriminator.

In J-RESRGAN, we introduce a fourth loss function, the symmetric loss, which exploits the vertical symmetry of J-RES NMR spectra (see main text for details).

In the training of Real-ESRGAN, four NIVDIA V100 GPUs are utilized, employing a total batch size of 48. The model is run for $400K$ iterations, with an initial learning rate as 1×10^{-4}.

1.2 Standards Database: NMR Data Acquisition

55 pure compound reagents were dissolved in water at various concentrations (Please see Table S1 & S2 for full list). NMR samples were prepared in 5 mm tubes by adding 540 μL of the previously prepared standard solution and 60 μL of D_2O buffer containing 1.5 M of KH_2PO_4, 5.8 mM of TSP (3-(trimethylsilyl)-2,2,3,3-tetradeuteropropionic acid or TMSP-d4) and 2 mM of NaN_3 at pH 7.4 [1].

1D-NOESY presat pulse sequence and J-Res 2D experiments were run in automation at 300 K in a Bruker Avance III HD 600 spectrometer working at 14.1 T equipped with a BBI probe. 32 Free Induction Decays (FID) were accumulated for each experiment in 64 K points using a 20-ppm window centered at 4.78 ppm while 2 scans of 8K points and 40 transients were acquired for the 2D J-Resolved experiment. The relaxation delay was set at 4 and 2 s respectively, and

a water pre-saturation pulse was applied during this period to cancel the water signal. Free induction decays of all 1D-spectra were multiplied by an exponential function equivalent to 0.3 Hz line-broadening before applying Fourier transform. Processing of the spectra (including Fourier transformation, phasing, baseline correction and calibration) was done in automation using Topspin 3.6 (Bruker Corporation, Germany).

Table S1. Concentrations of common urine metabolites used for simulating training J-Res NMR Spectra. This table lists the concentrations (mean and standard deviation, in micromolar units) for selected metabolites frequently found in normal adult human urine, each derived from averaging numerous concentration records per metabolite in the Human Metabolome Database (HMDB 5.0). The metabolites are ordered by their mean concentration values. This data was used to construct 5 distinct types of mixtures based on concentration thresholds: mixtures with mean concentrations greater than 1000 µM (mix_6), less than 1000 µM (mix_34), less than 100 µM (mix_15), less than 50 µM (mix_8), and a mixture encompassing all listed metabolites (mix_40).

No.	Metabolite Name	HMDB ID	Concentration Mean (µM)	Concentration STD (µM)
**1**	Creatinine	HMDB0000562	11995.25	7793.5
**2**	Hippuric Acid	HMDB0000714	3207.53	887.76
**3**	Citric Acid	HMDB0000094	3038.16	569.78
**4**	Creatine	HMDB0000064	1890.33	687.31
**5**	Glycine	HMDB0000123	1751.31	315.09
**6**	Trimethylamine N-Oxide	HMDB0000925	1257.70	435.66
**7**	Taurine	HMDB0000251	786.41	211.98
**8**	Histidine	HMDB0000177	711.44	130.46
**9**	L-Lactate	HMDB0000190	613.80	174.96
**10**	Phenylacetylglutamine	HMDB0006344	607.92	79.34
**11**	Formate	HMDB0000142	510.88	146.21
**12**	Isocitric Acid	HMDB0000193	427.39	82.77
**13**	L-Glutamine	HMDB0000641	401.12	63.98
**14**	Trigonelline	HMDB0000875	373.05	134.83
**15**	Betaine	HMDB0000043	366.45	70.38
**16**	L-Serine	HMDB0000187	330.71	46.92
**17**	Dimethylamine	HMDB0000087	323.87	22.52
**18**	L-Alanine	HMDB0000161	298.20	47.85
**19**	L-Lysine	HMDB0000167	264.14	43.88
**20**	D-Arabitol	HMDB0000182	229.47	57.59
**21**	p-Hydroxyphenylacetic Acid	HMDB0000568	227.91	46.76

**22**	Cis-aconitic Acid	HMDB0000020	195.76	42.94
**23**	Butyric Acid	HMDB0000072	176.69	62.27
**24**	L-Tyrosine	HMDB0000039	174.89	27.51
**25**	DL-Citrulline	HMDB0000904	117.67	25.72
**26**	D-Galactose	HMDB0000143	78.33	25.10
**27**	Succinic Acid	HMDB0000254	72.09	21.67
**28**	L-Leucine	HMDB0000687	69.69	9.04
**29**	L-Glutamic Acid	HMDB0000148	66.45	15.04
**30**	Trimethylamine	HMDB0000906	61.18	41.23
**31**	1-Methylnicotinamide	HMDB0000699	57.58	11.38
**32**	Dimethylglycine	HMDB0000092	56.02	12.63
**33**	Phenylacetic Acid	HMDB0000209	49.90	7.09
**34**	L-Carnosine	HMDB0000033	40.18	9.82
**35**	L-Isoleucine	HMDB0000172	35.27	6.70
**36**	2-Hydroxybutyric Acid	HMDB0000008	33.59	7.40
**37**	N-Acetyl-L-Aspartic Acid	HMDB0000812	26.39	5.38
**38**	L-Methionine	HMDB0000696	18.59	4.05
**39**	p-Cresol Sulfate	HMDB0011635	15.59	6.78
**40**	2-DeoxyAdenosine	HMDB0000101	2.64	0.70

Table S2. Concentrations and spectral complexity of key metabolites in human blood plasma for testing J-Res NMR spectra simulation. This table presents the concentrations (mean and standard deviation, in micromolar units) for metabolites frequently found in normal adult human blood plasma, each derived from averaging numerous concentration records per metabolite in the Human Metabolome Database (HMDB 5.0). Additionally, it includes the number of non-zero pixels, reflecting the complexity of their J-Res NMR spectra. The metabolites are organized based on the increasing complexity of their spectra, as indicated by the number of non-zero pixels (out of 256×16,384 pixels. Based on this complexity, 6 types of mixtures (mix_1, mix_3, mix_6, mix_9, mix_12, mix_15) are constructed sequentially from the table (i.e. least complexity, lowest 3 complexities, etc.), enabling the generation of simulated J-Res spectra with varying levels of complexity for model testing.

No.	Metabolite Name	HMDB ID	Concentration Mean (μM)	Concentration STD (μM)	Nonzero Pixels
**1**	L-Lactate	HMDB0000190	2011.80	734.29	1083
**2**	L-Threonine	HMDB0000167	147.70	20.80	1177
**3**	3-Hydroxybutyric Acid	HMDB0000011	65.93	36.35	1303

**4**	L-Valine	HMDB0000883	235.04	41.70	1490
**5**	L-Arginine	HMDB0000517	108.42	14.53	1823
**6**	L-Ornithine	HMDB0000214	76.86	18.58	2039
**7**	L-Proline	HMDB0000162	192.92	44.18	2975
**8**	1,6-Anhydro-beta-D-Glucose	HMDB0000640	200.00	20.00	3244
**9**	L-Tryptophan	HMDB0000929	65.49	7.39	3665
**10**	Glycerol	HMDB0000131	247.96	73.82	4548
**11**	L-Cysteine	HMDB0000574	74.47	9.25	4843
**12**	L-Phenylalanine	HMDB0000159	77.72	9.30	5406
**13**	alpha-D-Glucose	HMDB0003345	200.00	20.00	7564
**14**	Glucose	HMDB0000122	4908.19	428.96	8172
**15**	Methyl-hexadecanoic Acid	HMDB0061859	200.00	20.00	8991

1.3 Pre-processing Spectra for Standard Pure Compound and Experimental Mixtures

In the pre-processing of standard pure compound spectra, several crucial steps were undertaken to ensure data integrity and quality. Initially, we removed specific regions possibly containing calibration peaks and the water suppressed signal. To further enhance the clarity of each spectrum, we filtered noise with a threshold at 0.05 times the maximum value of the spectrum, thereby effectively minimizing background interference. Additionally, each spectrum was smoothed using the $signal.convolve2d$ function with a 3×3 window. Lastly, to facilitate comparisons between spectra, normalization was conducted. This was achieved by dividing each spectrum by the sum of all its values.

The pre-processing methodology for experimental mixture spectra mirrored that of the standard pure compound spectra. This included the critical steps of removing calibration peaks and water suppression, alongside smoothing spectra, and normalization.

1.4 Simulated Mixture Spectra

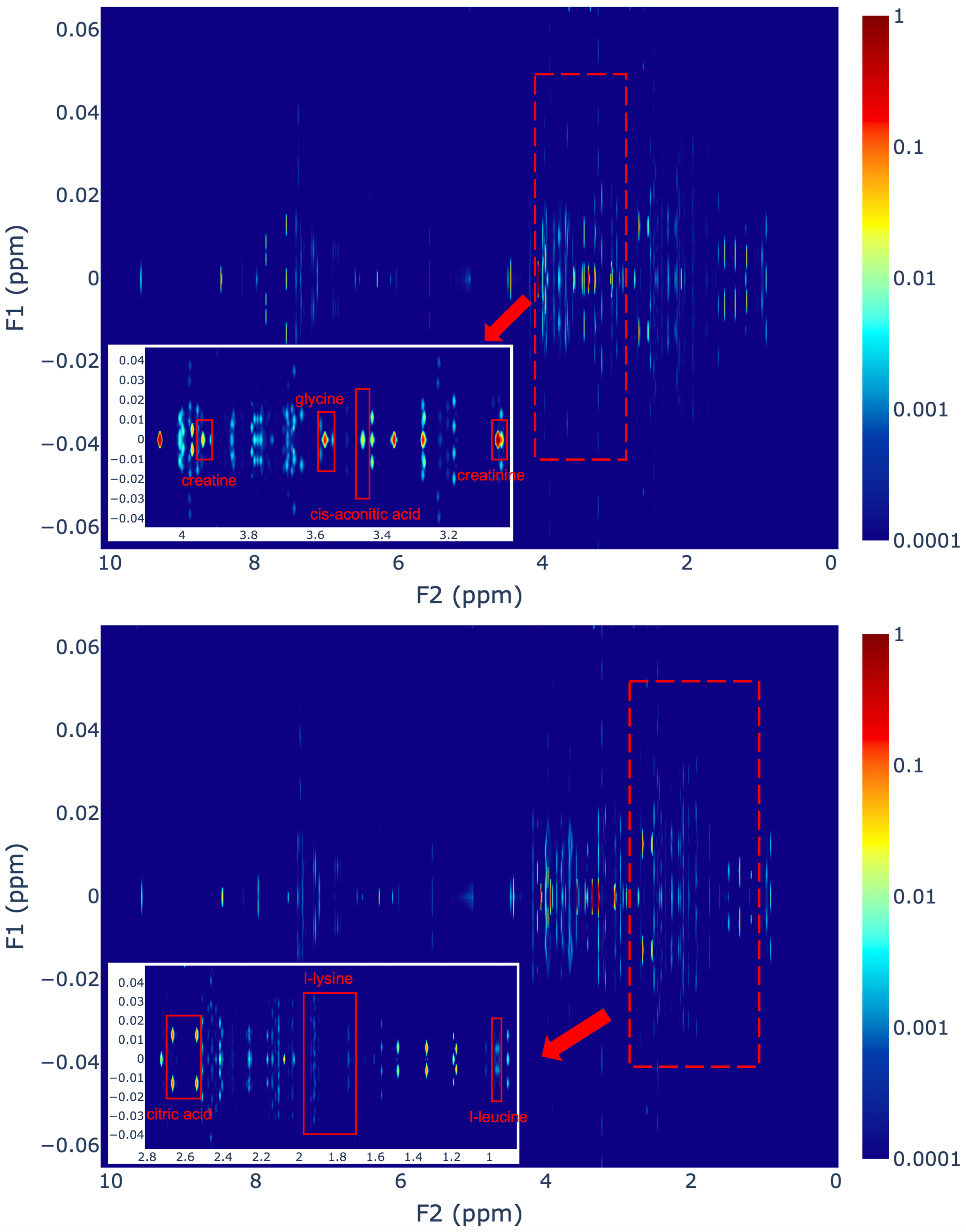

Figure S1. Examples of **Simulated** 2D J-Res NMR spectra for human **urine** mixtures. The two spectra are both simulated with all the 40 metabolites (Table S1) frequently found in human urine samples, differing only in the concentration levels of each metabolite. Insets highlight some specific metabolites. These spectra are normalized to a maximum intensity of one. Note the logarithmic color scale.

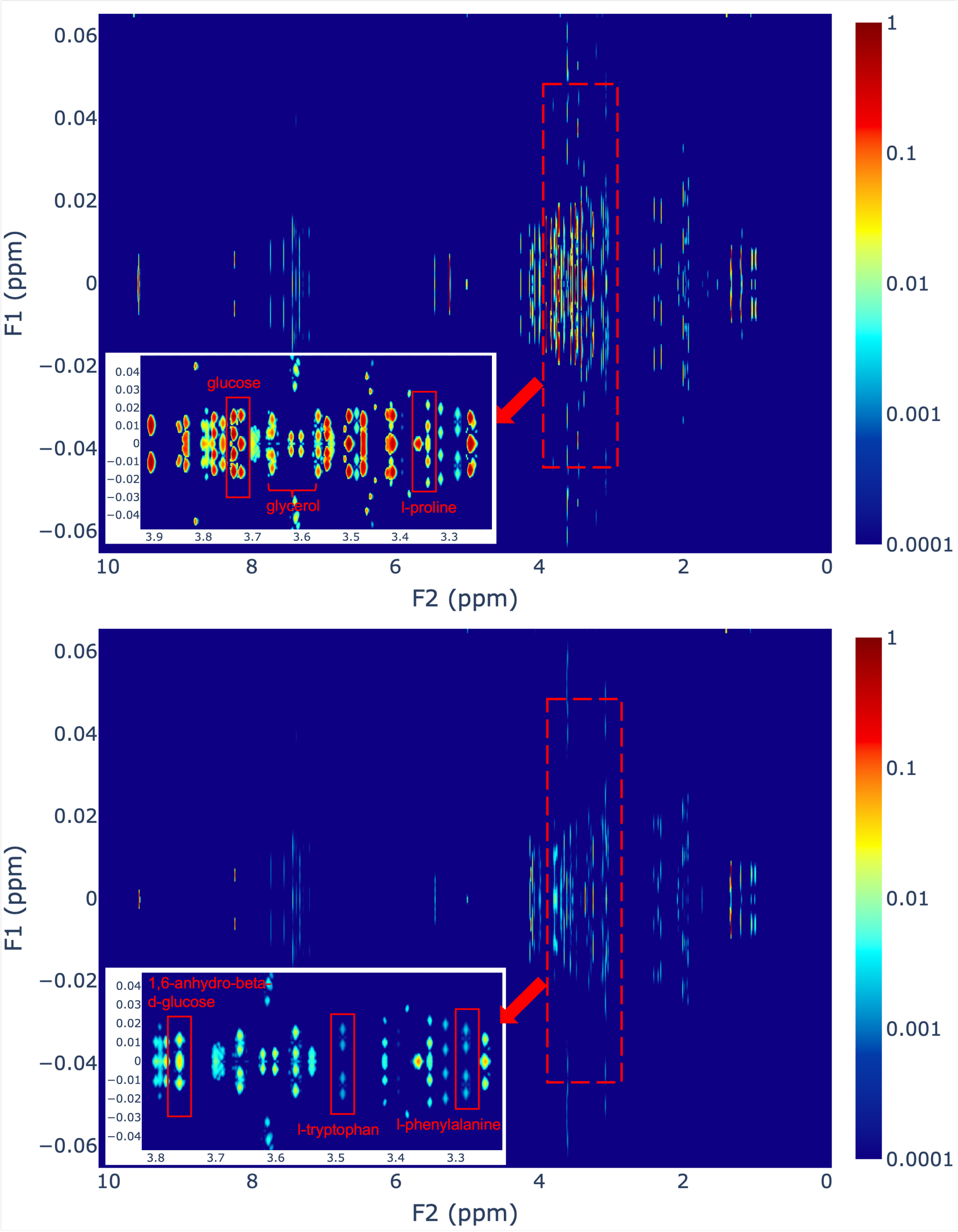

Figure S2. Examples of **simulated** 2D J-Res NMR spectra for human **plasma** mixtures. The upper panel displays a simulated spectrum with 15 metabolites (Table S2) frequently found in plasma samples. The lower panel shows a simulated spectrum with a subset of 12 metabolites from the original 15 metabolites, chosen based on their spectral complexity (Table S2). These spectra are normalized to maximum intensity of one. Note the logarithmic color scale.

1.5 Experimental J-Res Data of Complex Mixtures (plasma, urine, milk, orange juice)

The human blood plasma and urine samples were prepared as previously described in 5 mm NMR tubes [1]. In brief, 300 μL of plasma were mixed with 300 μL of buffer (75 mM of NaH_2PO_4, 4.6 mM of TSP (3-(trimethylsilyl)-2,2,3,3-tetradeuteropropionic acid or TMSP-d4) and 6.2 mM of NaN_3 at pH 7.4 in a 80:20 H_2O:D_2O mix). Urine samples were prepared by adding 540 μL and 60 μL of D_2O buffer containing 1.5 M of KH_2PO_4, 5.8 mM of TSP (3-(trimethylsilyl)-2,2,3,3-tetradeuteropropionic acid or TMSP-d4) and 2 mM of NaN_3 at pH 7.4. Milk was used as another example of protein containing complex mixture and was prepared following the plasma/serum protocol. Orange juice was used as an example of a non-proteinic complex mixture and was prepared following the urine protocol.

NMR spectra were acquired on a Bruker Avance III 600 MHz spectrometer (Bruker Biospin Ltd., Germany) operating at 600 MHz and 310 or 300 K for proteinic or non-proteinic samples, respectively. 2D J-Res experiments were acquired using the pulse sequence: -RD-90°-t1-180°-t1-ACQ, where RD is the relaxation delay (2 s), 90° represents the 90° radio frequency (RF) pulse, t1 is the increment delay and ACQ is the data acquisition period. Irradiation at the water resonance frequency using a series of 25 Hz RF pulses was applied during the RD. J-Res spectra were acquired using either 2 or 16 scans per increment over 40, 80 or 160 increments and either 8192, 16384 or 32768 data points in F2, using spectral widths of 16.6 ppm in F2 and 78 or 93 Hz in F1. Data was processed in automation using TopSpin v3.6. In order to obtain the same matrix dimension for the image processing, 16384 and 256 data points were used consistently across all the spectra. Fourier transform for the two dimensions using sine apodization functions and fsc mode (forward, single channel, complex), tilting and symmetrizing were applied, as well as baseline correction using polynomial of degree 5 (least square fit) quadrature, and 1 and 0.3 Hz line broadening in F2 and F1 respectively.

Using this experimental data we obtained 4 pairs of low resolution (LR) and high resolution (HR) spectra for blood samples, 5 pairs for urine (4 at 600 MHz and 1 at 800 MHz), 2 for milk and 2 for juice. Each (LR, HR) pair corresponds to spectra with different numbers of increments. For example, one pair could be (40, 80) increments while a second pair could be (80, 160) increments. We also analyzed one pair of LR and HR J-Res data for urine obtained on an 800 MHz Bruker spectrometer equipped with a TXI cryoprobe.

Below is a more detailed description of each set of spectra:

Plasma: two samples (plasma 1 and plasma 2), NS = 16

- sample 1: LR-8192 x 40, HR-8192 x 80, HHR-8192 x 160.
- sample 2: LR-8192 x 40, HR-8192 x 80, HHR-8192 x 160.

➔ get 4 pairs: (LR, HR), (HR, HHR) for each sample.

Urine: one sample (urine 1), NS = 16, processed at different spectral width (The different spectral widths are caused by the system updates of Bruker Topspin. We used these different spectral widths to explore response of J-RESRGAN to heterogeneity in data.)

- SW = 0.15: LR-8192 x 40, HR-16384 x 80, HHR-32768 x 160.
- SW = 0.13: LR-8192 x 40, HR-16384 x 80, HHR-32768 x 160

➔ get 4 pairs: (LR, HR), (HR, HHR) for each sample.

Urine at 800 MHz: one sample (urine 2)

- LR: 8192 x 40, NS = 1
- HR: 8192 x 128, NS = 16

➔ get one pair of LR and HR

Milk: one sample, NS = 8, at 600 MHz

- SW = 0.13: LR-8192 x 40, HR-16384 x 80, HHR-32768 x 160.

➔ get 2 pairs: (LR, HR), (HR, HHR)

Orange Juice: one sample, NS = 8, at 600 MHz

- SW = 0.13: LR-8192 x 40, HR-16384 x 80, HHR-32768 x 160.

➔ get 2 pairs: (LR, HR), (HR, HHR)

1.6 Experimental Pairs of Spectra for Comparison with NUS

To assess the capabilities of J-RESRGAN in enhancing spectral resolution, a comparative analysis was designed using six J-Res NMR spectra derived from one human urine sample (urine 3, different from the urine samples in section 1.5). This set included three spectra obtained via standard acquisition and three via non-uniform sampling (NUS), with experimental parameters detailed in the Table S3.

The three standard spectra, LR, HR, and HHR, were acquired as described in section 1.5. In contrast, the spectra with NUS acquisition were sampled at varying percentages to facilitate data collection while aiming to match the resolution of higher increment spectra. Specifically, NUS 1 achieved an HR-equivalent resolution (80 increments) with a 50% sampling rate, doubling the resolution relative to LR within a comparable acquisition time. NUS 2 got an HHR-equivalent resolution (160 increments) with a 50% sampling rate, doubling the resolution compared to HR. NUS 3 employed a 25% sampling rate to achieve HHR-level resolution (160 increments) within the time typically required for LR acquisition, yielding a 4-fold resolution enhancement (Table S3). All the spectra have been processed as described in section 1.5.

The analysis was based on three pairs:

Pair 1: LR, HR, with NUS 1 and J-RESRGAN applied to LR to generate a SR spectrum.

Pair 2: HR, HHR, with NUS 2 and J-RESRGAN applied to HR to generate a SR spectrum.

Pair 3: LR, HHR, with NUS 3 and J-RESRGAN applied to NUS 1 to generate s SR spectrum.

Table S3. Acquistion parameters in TopSpin for the acquisition of J-Res spectra of a human urine sample, utilizing both standard and non-uniform sampling (NUS) acquisition techniques. Notably, "TD" denotes the size of the FID; "SI" refers to the size of the processed spectrum; "SW" indicates the spectral width; and "NS" represents the number of scans.

Index	Spectral name	Increments	% NUS	TD	SI	SW	NS	Acquisition time

**1**	LR	40	/	8192 × 40	16384 × 256	0.13	2	~ 4 min
**2**	HR	80	/	8192 × 80	16384 × 256	0.13	2	~ 8 min
**3**	HHR	160	/	8192 × 160	16384 × 256	0.13	2	~ 19 min
**4**	NUS 1	80	50%	8192 × 80	16384 × 256	0.13	2	~ 4 min
**5**	NUS 2	160	50%	8192 × 160	16384 × 256	0.13	2	~ 9 min
**6**	NUS 3	160	25%	8192 × 160	16384 × 256	0.13	2	~ 4 min

1.7 Experimental Pairs of Spectra for Comparison with Linear Prediction

Two pairs of experimental LR and HR spectra derived from one plasma sample (plasma 3, different from the plasma samples in section 1.5) were used here to compare the efficiency of J-RESRGAN and linear prediction. The J-Res NMR spectra were acquired exactly the same as described in section 1.5. Below is more detailed description for the two pairs of spectra:

Pair 1: NS = 16, SW = 0.15, at 600 MHz

- LR: TD = 8192 x 40, SI = 16384 x 256
- HR: TD = 16384 x 80, SI = 16384 x 256

Pair 2: NS = 2, SW = 0.15, at 600 MHz

- LR: TD = 8192 x 40, SI = 16384 x 256
- HR: TD = 16384 x 80, SI = 16384 x 256

In brief, these two pairs are only different in the number of scans used during the acquisition process.

The linear prediction settings in TopSpin are shown in Table S3.

Table S4. The parameter settings in TopSpin for linear prediction to enhance the resolution of LR spectra.

Parameters for LP in TopSpin	Applied to F2 & F1	Note for setting
**ME_mod**	LPfc	Forward linear prediction on complex data

**NCOEF**	32	"32" is a standard, default value
**LPBIN**	0	"0" means 2-fold linear prediction

1.8 Selection of Optimal J-RESRGAN Model

The J-RESRGAN model, developed as a modification of Real-ESRGAN, retains several fundamental training parameters, including the learning rate and the weights assigned to the original loss functions. A notable adaptation in J-RESRGAN is the introduction of a novel symmetric loss function, assigned a weight of 2, underscoring its importance in the model training. Consequently, J-ESRGAN comprises symmetric loss, pixel loss, perceptual loss, and GAN loss weighted at {2, 1, 1, 0.1}, respectively.

The training process encompassed approx. 86,000 iterations, with the model's efficacy evaluated against the aggregated loss functions, as shown in Figure S3. e). The trend of the aggregated loss revealed an optimal performance of the generator model at approximately 45,000 iterations, characterized by the lowest loss value. Thus, this iteration was selected as the most effective model for subsequent super-resolution spectra generation tasks.

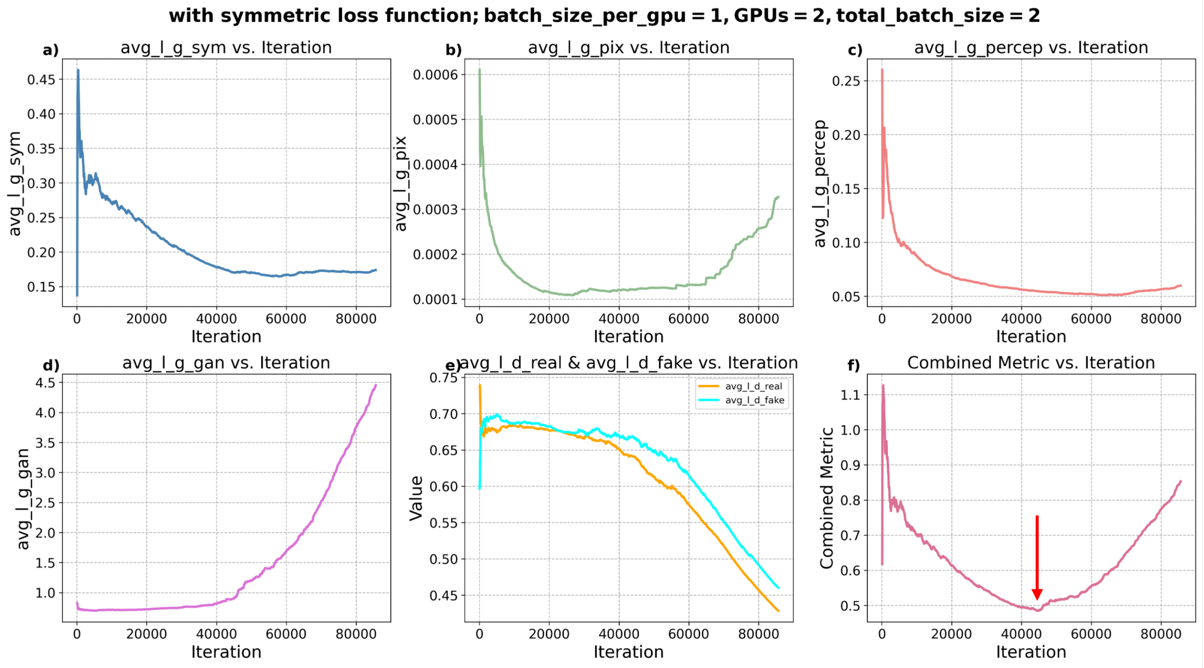

Figure S3. Training dynamics of J-RESRGAN. Subplot a) shows the symmetric loss function, weighted by 2. Subplot b) and c) depict the pixel loss function and perceptual loss function, with a weight of 1, respectively. Subplot d) shows the loss of the generator, with a lower weight of 0.1, while subplot e) shows the loss of the discriminator when assessing real (avg_l_d_real) versus generated (avg_l_d_fake) images. Subplot f) combines all the loss functions (subplot a), b), c), d)) used in the training of the network into a single metric, offering an overall view of the model's performance.

1.9 Resolvability Score Plot

As discussed in the main text, we introduce a score which quantifies how well two overlapping peaks can be resolved:

$$resolvability\ score = 1 - \frac{h_v}{\min(h_i, h_j)}$$

where h_v, h_i, and h_j symbolize the heights of the valley floor and the respective heights of two adjacent peaks.

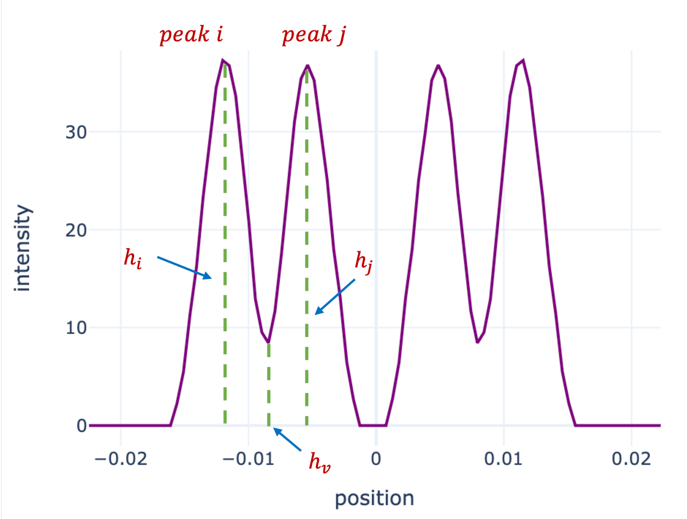

Figure S4. An example peak pair for calculating resolvability scores. h_v, h_i, and h_j symbolize the heights of the valley floor and the respective heights of two adjacent peaks.

2. Results

2.1 Peak Intensity vs. Resolvability

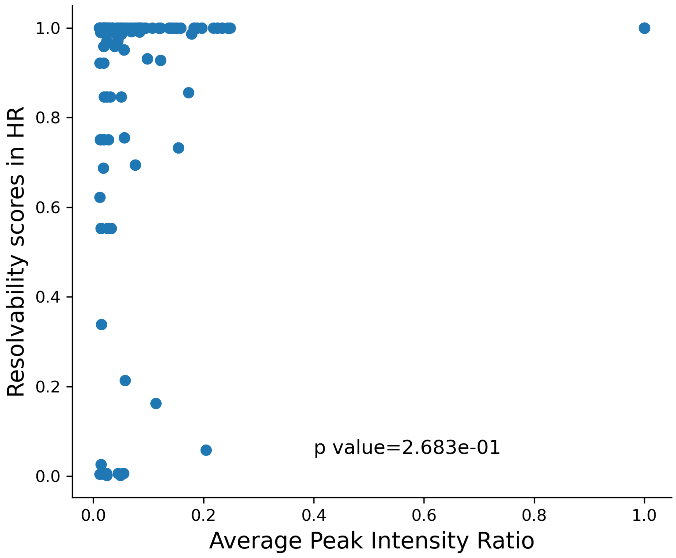

Figure S5. The relationship between averaged peak intensity ratios and resolvability scores in HR.

2.2 Experimental Mixture Spectra

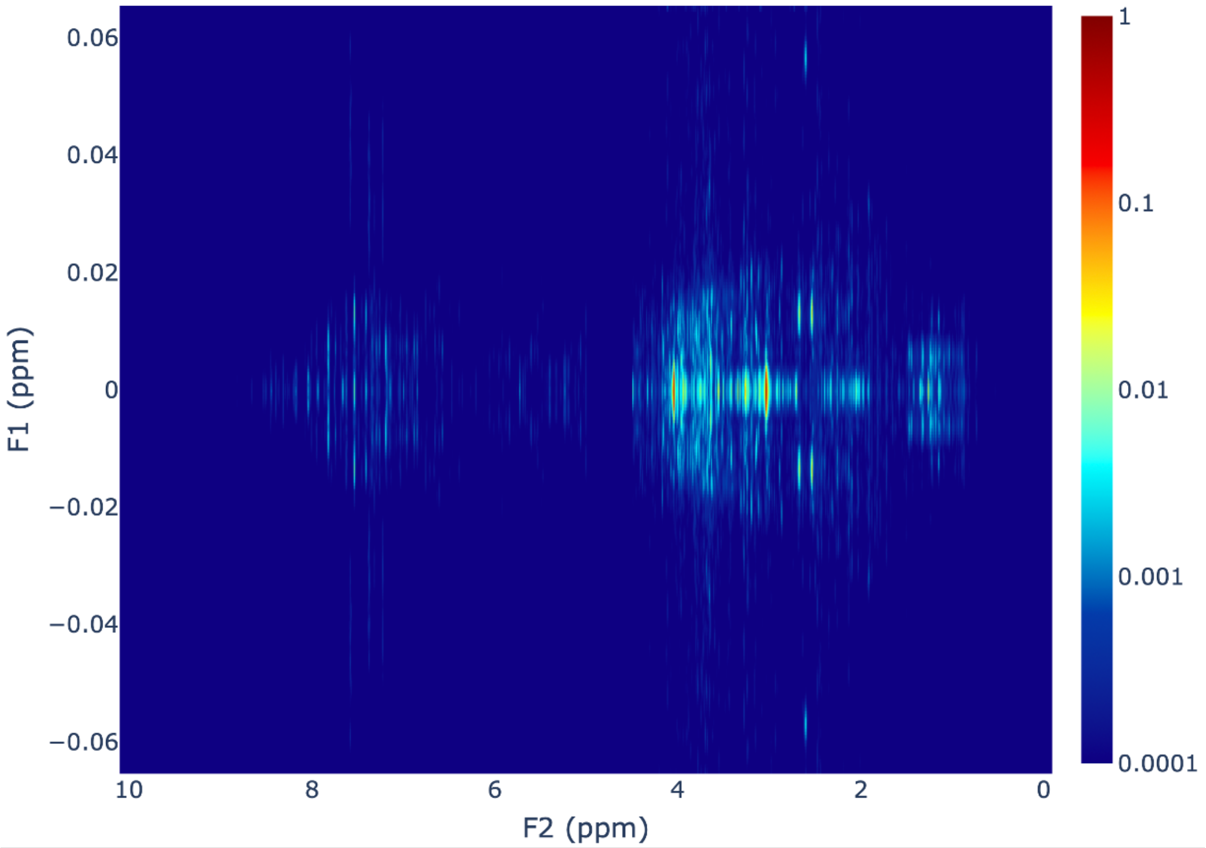

Figure S6. Example of **experimental** 2D J-Res NMR spectrum of human **urine**, urine 1, with certain pre-processing steps including removing internal standard and water suppression residual, removing noise using a threshold set at 10 times noise standard deviation (for presenting only), smoothing, and normalizing to the maximum value. Note logarithmic color scale.

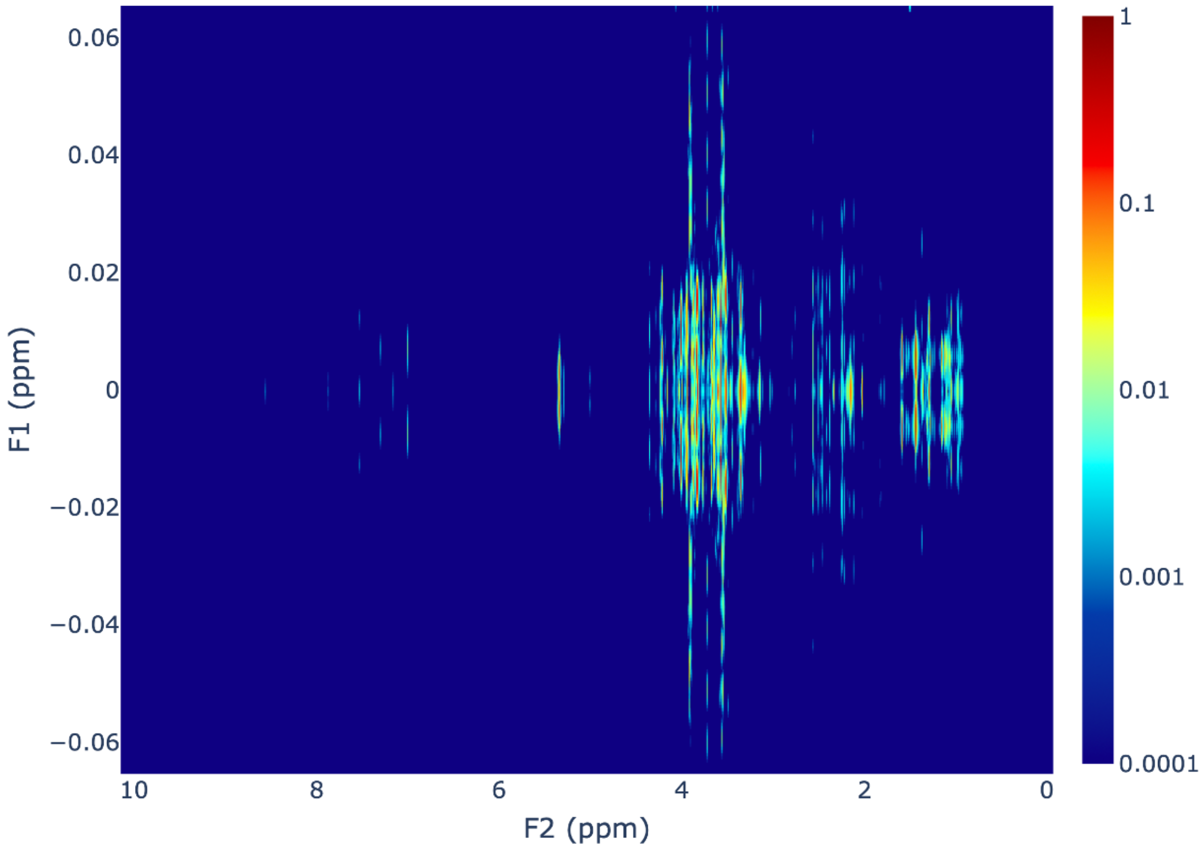

Figure S7. Example of **experimental** 2D J-Res NMR spectrum of human **plasma**, plasma 1, with certain pre-processing steps including removing internal standard and water suppression residual, removing noise using a threshold set at 10 times noise standard deviation (for presentation only), data smoothing, and normalizing to the maximum value. Note the logarithmic color scale.

2.3 Matching Peaks between HR and SR Spectra

Algorithm 1 Matching Peaks between HR and SR Spectra

Input HR and SR spectra: im_hr, im_sr
Output peak pair profiles of SR

```
procedure
    Resize the HR data to match the size of the SR data
    Peak Picking on HR with a threshold, e.g., 0.01
    Peak Picking on SR with a threshold, e.g., 0.01
    for the position of each picked peaks in HR, p_hr do
        if matching with p_sr within a 2-pixel tolerance at F2 scale and a 4-pixel tolerance at F1 scale then
            use the positions of SR for this peak
        else if not matching then
            use the positions of HR for this peak
        end if
    end for
    Get the sliced 1D profiles for each peak pair in SR
end procedure
```

2.4 V Plot for Plasma Samples at 600 MHz

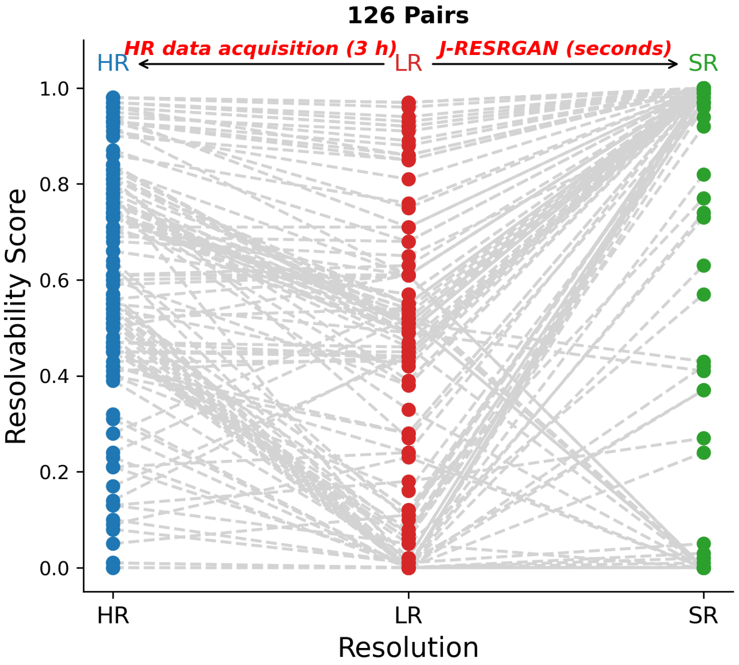

Figure S8. Resolvability scores for matched peak pairs in HR, LR, and SR spectra of **plasma** mixtures. This figure displays the resolvability scores for 3 additional pairs of LR and HR spectra. The pairs of spectra collectively have detection of 47, 27, 52 peak pairs, respectively, with each pair demonstrating a doubled number of pixels along the F1 axis. Among these, 87.2% of the 47 peak pairs, 100% of the 27 peak pairs, and 80.8% of the 52 peak pairs exhibit enhanced resolution in the SR compared to the LR.

2.5 V Plot for Urine Samples at 600 MHz

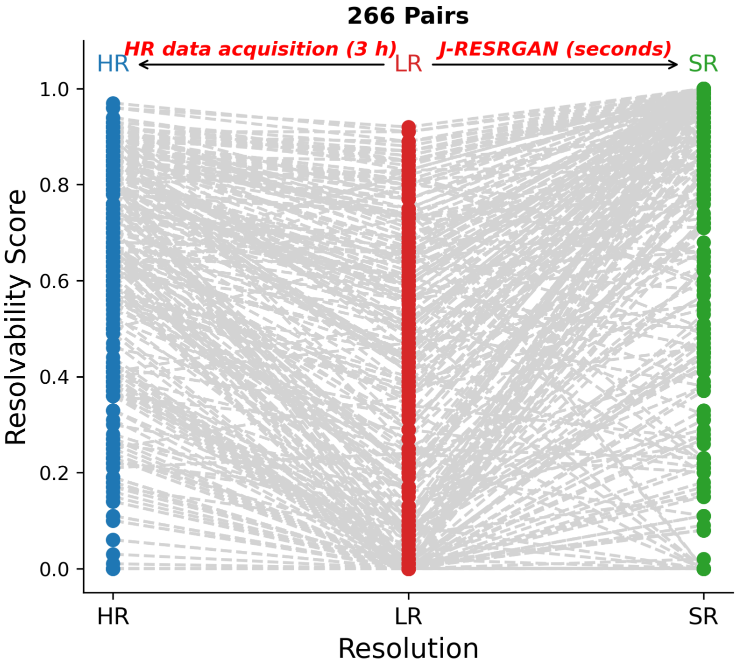

Figure S9. Resolvability scores for matched peak pairs in HR, LR, and SR spectra for **urine** mixtures. This figure displays the resolvability scores for 4 pairs of LR and HR spectra. The pairs of spectra collectively have detection of 75, 101, 70, 20 peak pairs, respectively, with each pair demonstrating not only a doubled number of pixels along the F1 axis, but also a doubled

number of pixels along the F2 axis. Among these, 90.7% of the 75 peak pairs, 85.1% of the 101 peak pairs, 90.0% of the 70 peak pairs, and 85.0% of the 20 peak pairs exhibit enhanced resolution in the SR compared to the LR.

2.6 V Plot for Urine Samples at 800 MHz

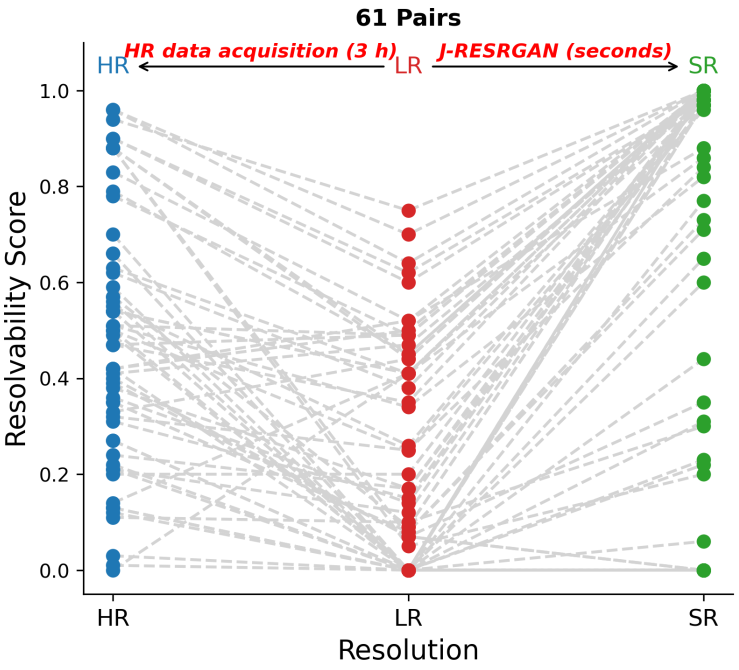

Figure S10. Resolvability scores for matched peak pairs in HR, LR, and SR spectra of **urine** mixtures acquired at **800 MHz**. This figure displays the resolvability scores for 1 pair of LR and HR spectra. The pair of spectra collectively have detection of 61 peak pairs, with the HR exhibiting approximately 3 times the number of pixels along the F1 axis compared to the LR spectrum. There are 96.7% of the 61 peak pairs exhibiting enhanced resolution in the SR compared to the LR.

2.7 V plot for Milk at 600 MHz

Example of J-RESGAN performance on a proteinic sample different from human blood plasma.

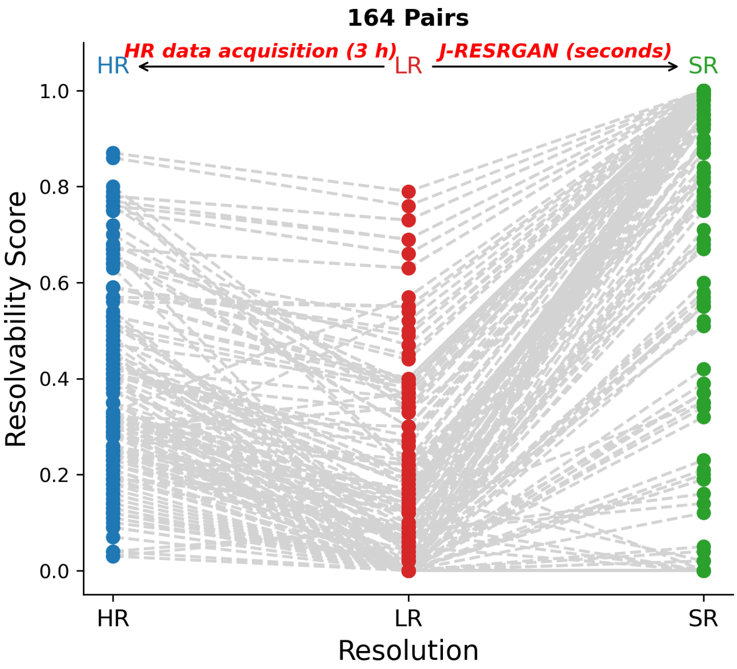

Figure S11. Resolvability scores for matched peak pairs in HR, LR, and SR spectra of **full fat milk**. This figure displays the resolvability scores for 2 pairs of LR and HR spectra. The pairs of spectra collectively have detection of 93, 71 peak pairs, respectively, with each pair demonstrating not only a doubled number of pixels along the F1 axis, but also a doubled number of pixels along the F2 axis. Among these, 98.9% of the 93 peak pairs, and 94.4% of the 71 peak pairs show enhanced resolution in the SR compared to the LR.

2.8 V Plot for Orange Juice at 600 MHz

Example of J-RESGAN performance on a non-proteinic sample different from human urine.

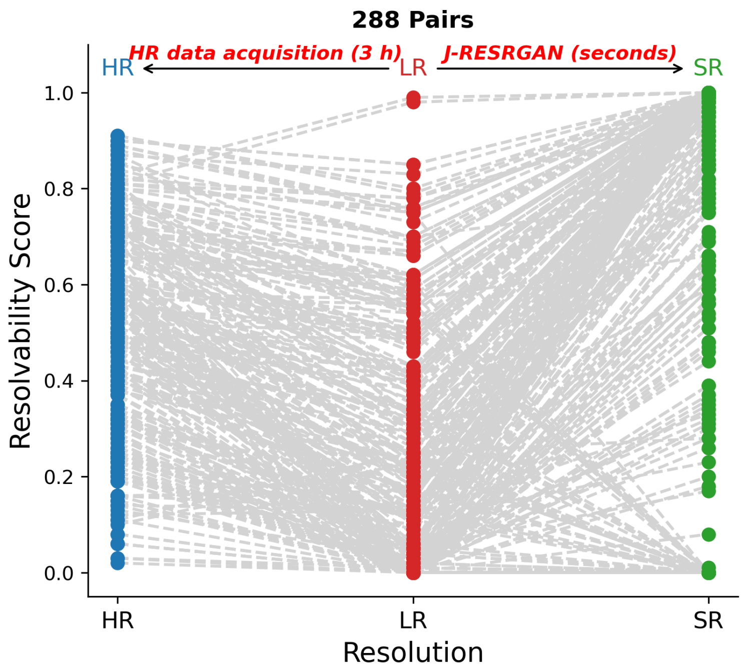

Figure S12. Resolvability scores for matched peak pairs in HR, LR, and SR spectra of **orange juice**. This figure displays the resolvability scores for 2 pairs of LR and HR spectra. The pairs of spectra collectively have detection of 144, 144 peak pairs, respectively, with each pair demonstrating not only a doubled number of pixels along the F1 axis, but also a doubled number of pixels along the F2 axis. Among these, 91.7% of the 144 peak pairs, and 82.6% of the 144 peak pairs show enhanced resolution in the SR compared to the LR. There are a small number of peak pairs where resolvability scores in LR are larger than those in HR. This is attributed to the variance in background noise levels within the experimental spectra, leading to different valley heights and resolvability scores.

2.9 Examples of Poorly Resolved & Over Resolved Peak Pairs

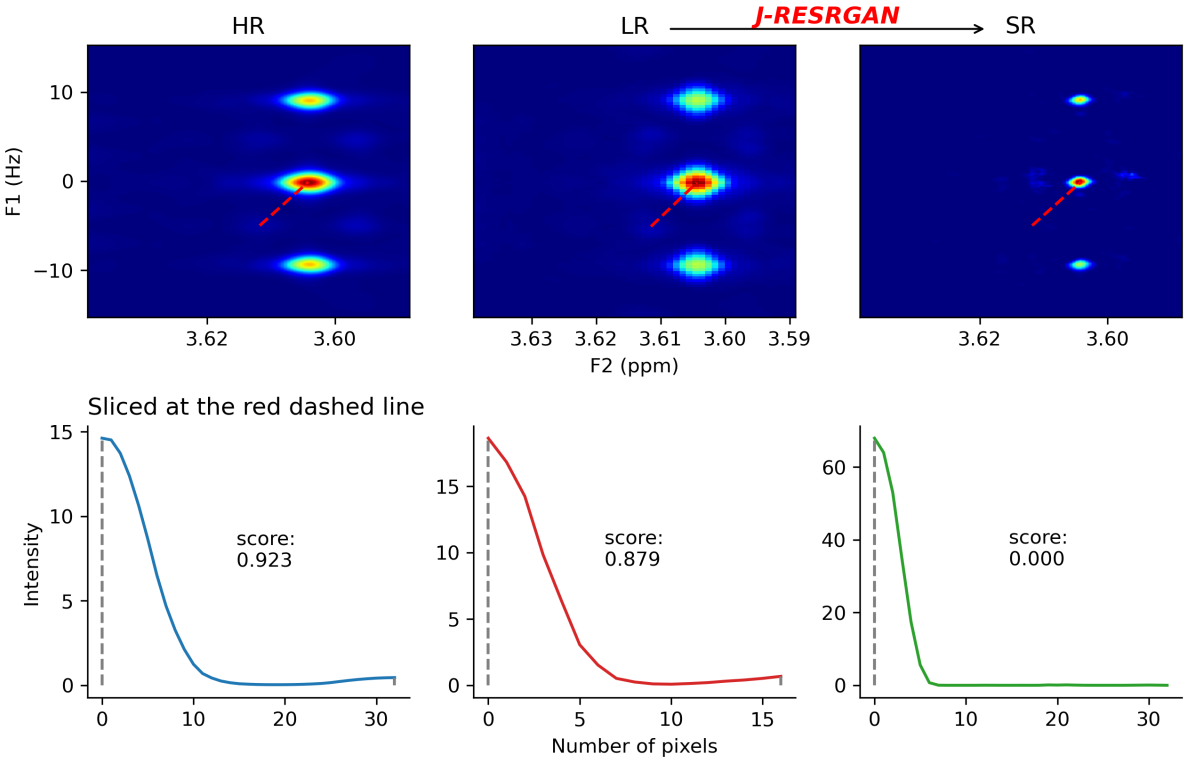

Figure S13. An example of poorly resolved peak pair in SR from experimental plasma data. Red dashed line in upper panels shows line between peak maxima, along which profiles in bottom panel are calculated. Some suppression of small peaks in the SR output can be observed.

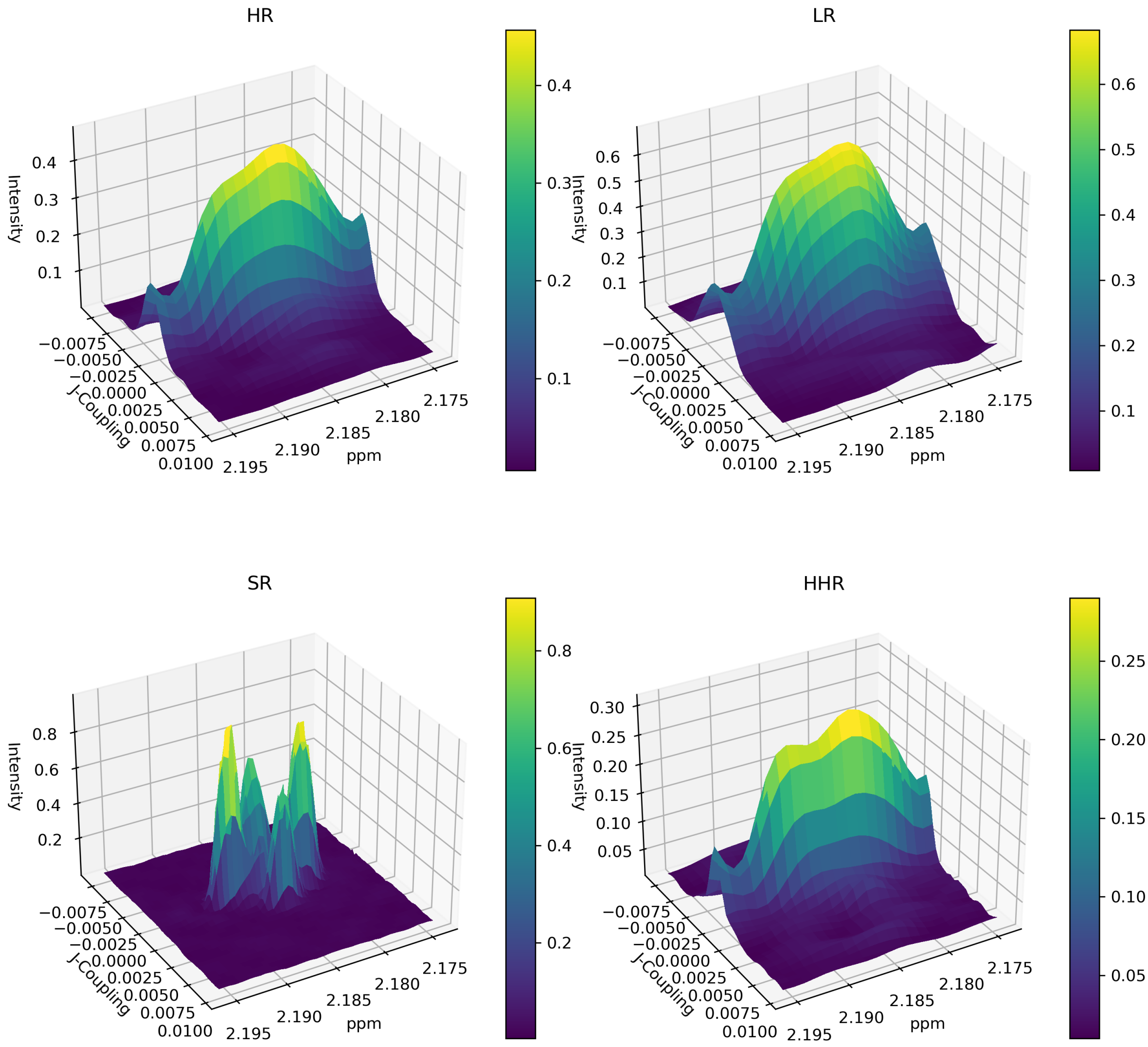

Figure S14. An example of over-resolved peak pair in SR from experimental plasma data. The SR estimated from LR input clearly shows several peaks at a higher apparent resolution than the HR or HHR data.

2.10 Comparison between J-RESRGAN and NUS

This section presents the results of a comparative analysis between non-uniform sampling (NUS) and J-RESRGAN, delineated through three illustrative examples (Figure S15, S16, and S17). Figure S15 and S16 demonstrate the superior capability of J-RESRGAN in resolving overlapping peaks. Notably, in the second example (Figure S16), J-RESRGAN outperforms NUS with statistical significance. The third example (Figure S17) provides evidence that the combination of NUS with J-RESRGAN significantly surpasses the performance of NUS alone in enhancing spectral resolution. The findings underscore J-RESRGAN's proficiency in enhancing spectra via standard acquisition, as well as those via traditional resolution-boosting

techniques. This illustrates the adaptability and efficiency of J-RESRGAN in a range of spectral acquisition scenarios. The same human urine sample was used for all these comparisons.

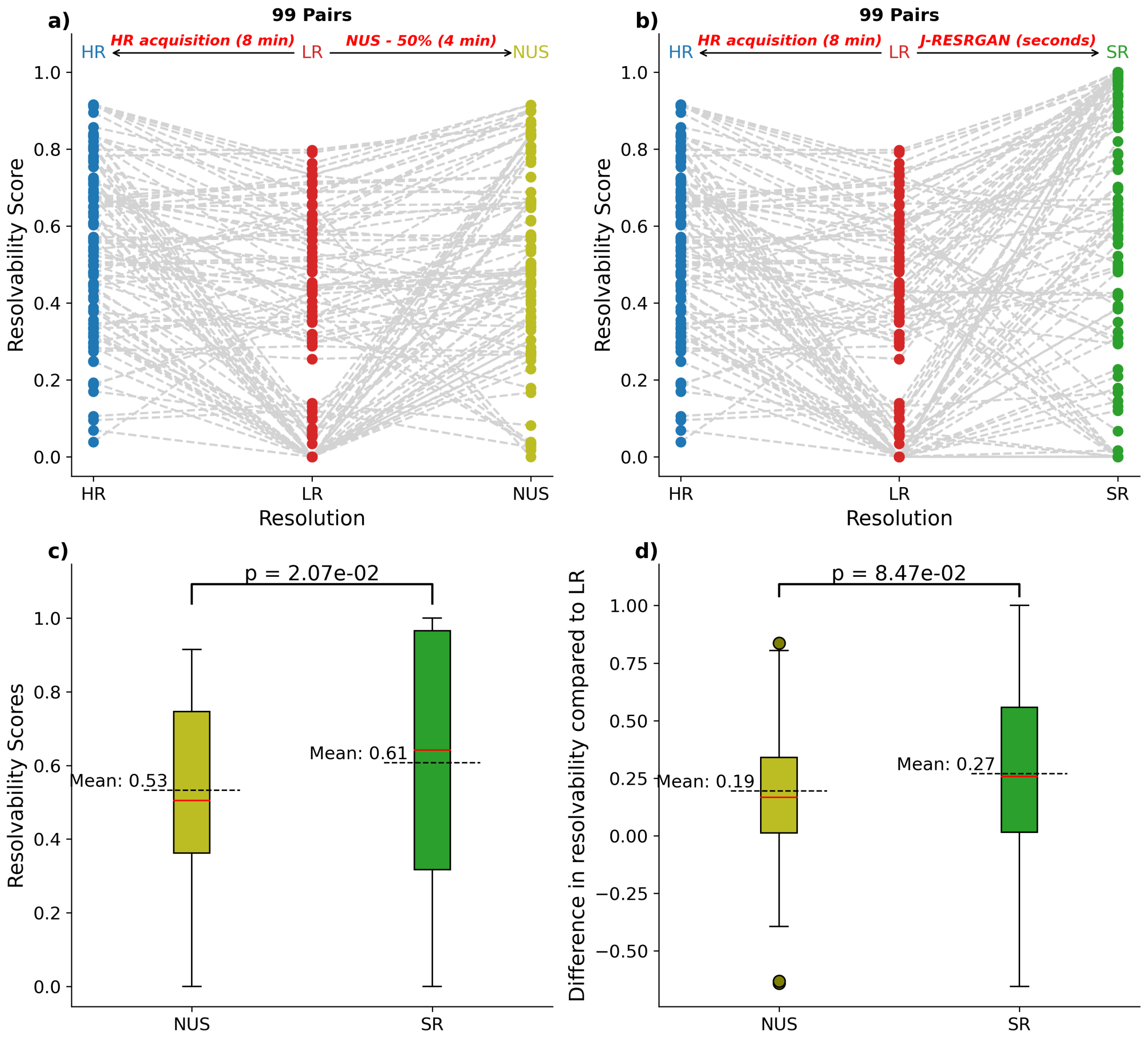

Figure S15. Comparative analysis of resolution enhancement methods: NUS and J-RESRGAN. J-RESRGAN was applied to the LR spectrum (40 increments) acquired from one urine sample with standard acquisition, effectively doubling the resolution. NUS achieved a HR-level resolution (80 increments) with a 50% sampling rate, mirroring the resolution doubling of LR within the same acquisition time. The resultant spectra from J-RESRGAN and NUS are denoted as SR and NUS, respectively. All spectra, LR, HR, SR, and NUS, were subject to the same level of smoothing to facilitate peak detection during resolvability score calculation. Plot a) illustrates resolvability scores for 99 peak pairs across HR, LR and NUS spectra. Plot b) displays resolvability scores for the same peak pairs in HR, LR, and SR spectra. Plot c) presents box plots comparing the resolvability scores obtained by NUS and SR, with the SR showing higher median resolvability. Plot d) shows box plots of the difference of resolvability scores relative to LR for both NUS and SR, with SR providing a more pronounced improvement in peak separation as indicated by higher median and mean scores. Statistical significance is calculated via a Mann Whitney test, with p = 2.07e-02 for NUS versus SR comparison and p = 8.47e-02 for the score difference comparison.

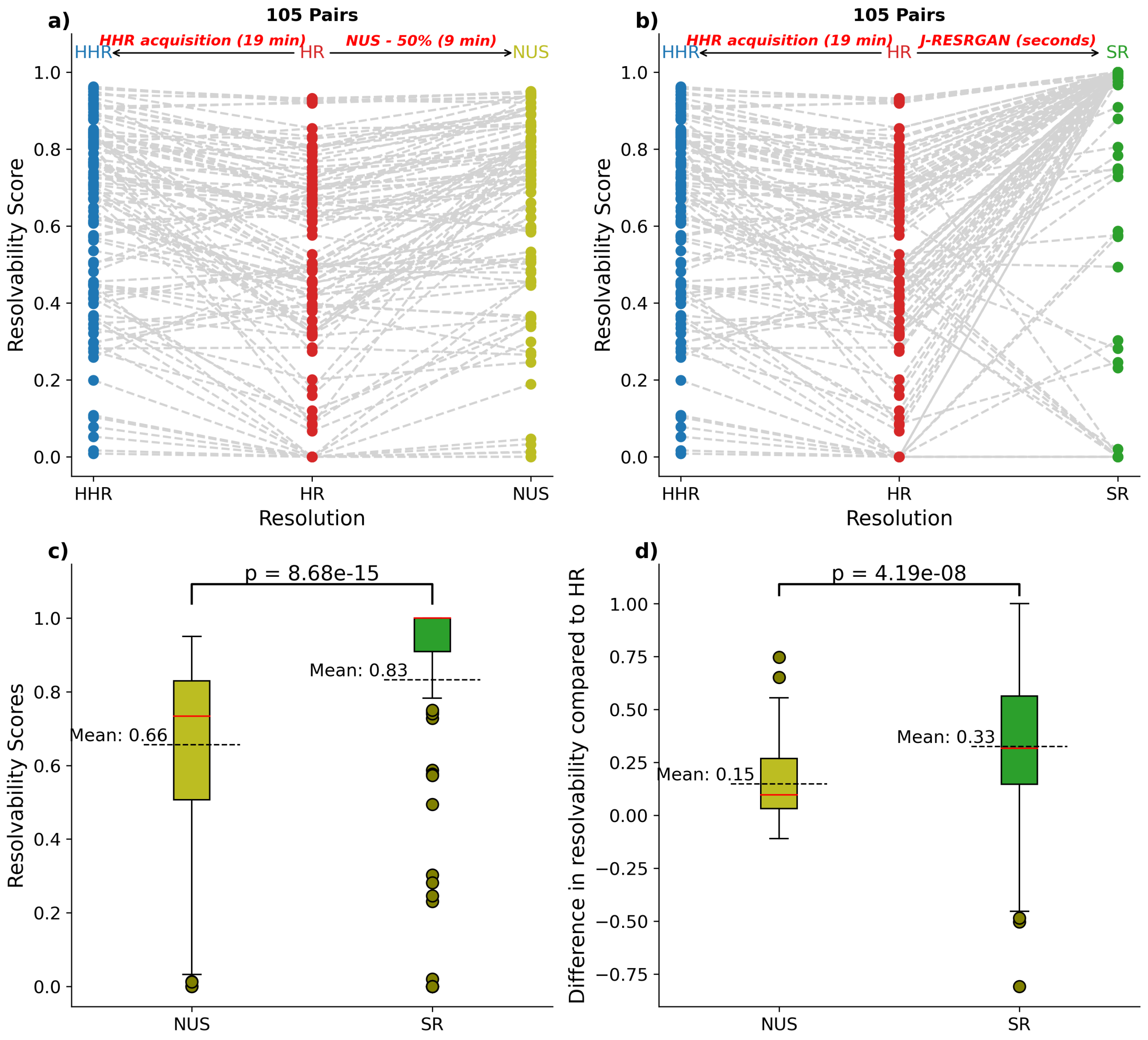

Figure S16. Comparative analysis of resolution enhancement methods: NUS and J-RESRGAN. J-RESRGAN was applied to the HR spectrum (80 increments) acquired from one urine sample with standard acquisition, effectively doubling the resolution. NUS achieved an HHR-level resolution (160 increments) with a 50% sampling rate, mirroring the resolution doubling of HR within the same acquisition time. The resultant spectra from J-RESRGAN and NUS are denoted as SR and NUS, respectively. All spectra, HR, HHR, SR, and NUS, were subject to the same level of smoothing to facilitate peak detection during resolvability score calculation. Plot a) illustrates resolvability scores for 105 peak pairs across HHR, HR and NUS spectra. Plot b) displays resolvability scores for the same peak pairs in HHR, HR, and SR spectra. Plot c) presents box plots comparing the resolvability scores obtained by NUS and SR, with the SR showing higher median and mean resolvability scores. Plot d) shows box plots of the difference of resolvability scores relative to HR for both NUS and SR, with SR providing a more pronounced improvement in peak separation as indicated by higher median and mean scores. Statistical significance is calculated via a Mann Whitney test, with p = 8.68e-15 for NUS versus SR comparison and p = 4.19e-08 for the score difference comparison.

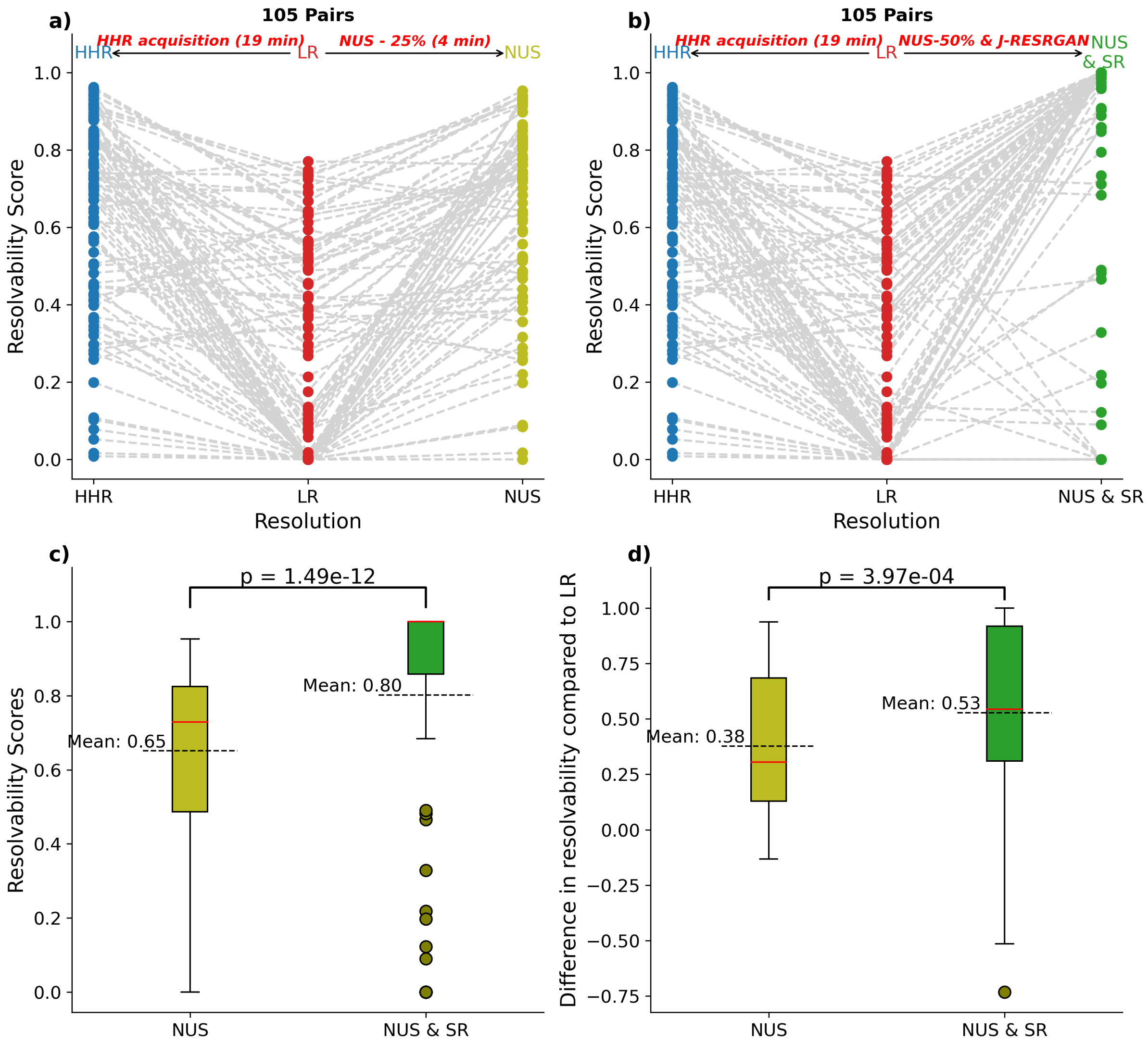

Figure S17. Comparative analysis of resolution enhancement methods: NUS alone and combining NUS and J-RESRGAN. These experimental J-Res spectra were derived from one urine sample. The spectrum acquired via NUS alone was denoted as NUS, while the spectrum generated through the combined approach of NUS followed by J-RESRGAN was labeled as NUS & SR. In plot a), the NUS spectrum achieved an HHR-level resolution (160 increments) with a 25% sampling rate, reflecting a 4-fold resolution increase compared to the LR within an equivalent acquisition time. A total of 105 peak pairs were analyzed across HHR, LR, and NUS spectra. In plot b), the NUS spectrum achieved a HR-level resolution (80 increments) with a 50% sampling rate, doubling the resolution relative to the LR. Subsequently, J-RESRGAN was applied to enhance this NUS spectrum further, ultimately reaching a 4-fold increase in resolution compared to LR. This plot displays resolvability scores for the same peak pairs in HHR, LR, and SR (applied to NUS) spectra. Plot c) presents box plots comparing the resolvability scores obtained by NUS alone and NUS combined with SR, revealing that the combined NUS & SR approach results in substantially higher median and mean resolvability scores. Plot d) features box plots of the differences in resolvability scores relative to LR for both NUS alone and NUS combined with SR, indicating that the integrated NUS & SR approach offers a more significant improvement in peak separation, as evidenced by the higher median and mean scores. The statistical significance of these comparisons was established using the Mann-Whitney test, yielding p-values of 1.49e-12 for the comparison between NUS alone and NUS & SR, and 3.97e-04 for the analysis of score differences.

2.11 Comparison between J-RESRGAN and linear prediction

This section delineates the results of a comparative analysis conducted between linear prediction and J-RESRGAN, utilizing two examples (Figure S18 and S19). The linear prediction approach was applied with parameters specified in Table S4. For the purpose of this study, forward linear prediction was employed due to its capability of enhancing resolution, in contrast to backward linear prediction which is typically reserved for data imputation [2]. A default number of coefficient of 32 was selected for application along both the F1 and F2 axes. In addition, to align with the resolution enhancement typically achieved by J-RESRGAN, which is generally a 2-fold increase, the "LPBIN" parameter was set to 0.

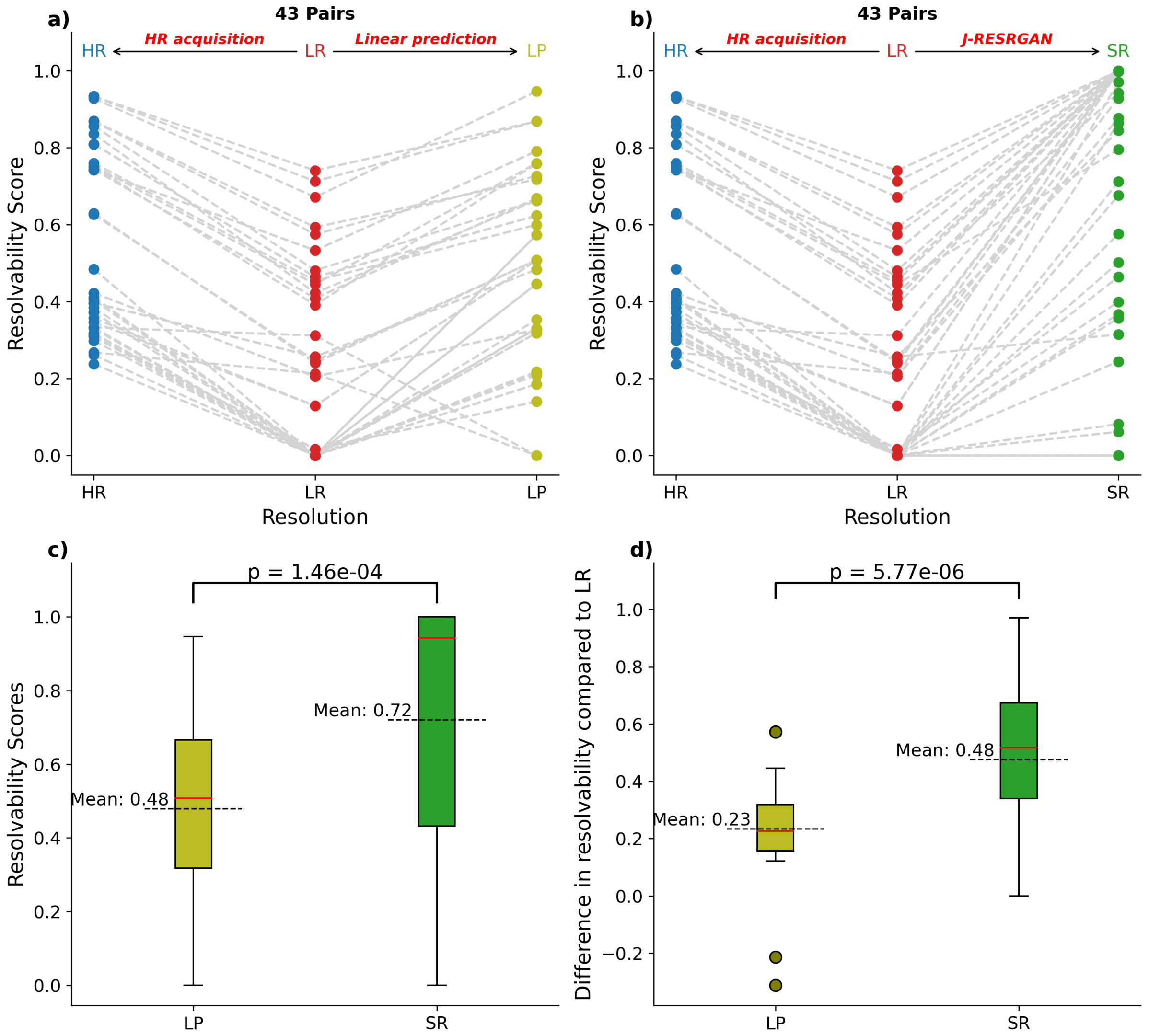

Figure S18. Comparative analysis of resolution enhancement methods: J-RESRGAN and linear prediction, applied to the LR spectrum acquired from a plasma sample with 16 scans (**NS = 16**). The spectra processed by J-RESRGAN and linear prediction are denoted as SR and LP, respectively. All spectra, LR, HR, SR, and LP, were subject to the same level of smoothing to facilitate peak detection during resolvability score calculation. Plot a) illustrates resolvability scores for 43 peak pairs across HR, LR and LP spectra. Plot b) displays resolvability scores for the same peak pairs in HR, LR, and SR spectra. Plot c) presents box plots comparing the resolvability scores obtained by LP and SR, with the SR showing higher median resolvability. Plot d) shows box plots of the difference of resolvability scores relative to LR for both LP and SR, with SR providing a more pronounced improvement in peak separation as indicated by higher median and mean scores. Statistical significance is calculated via a Mann Whitney test, with p = 1.46e-04 for LP versus SR comparison and p = 5.77e-06 for the score difference comparison.

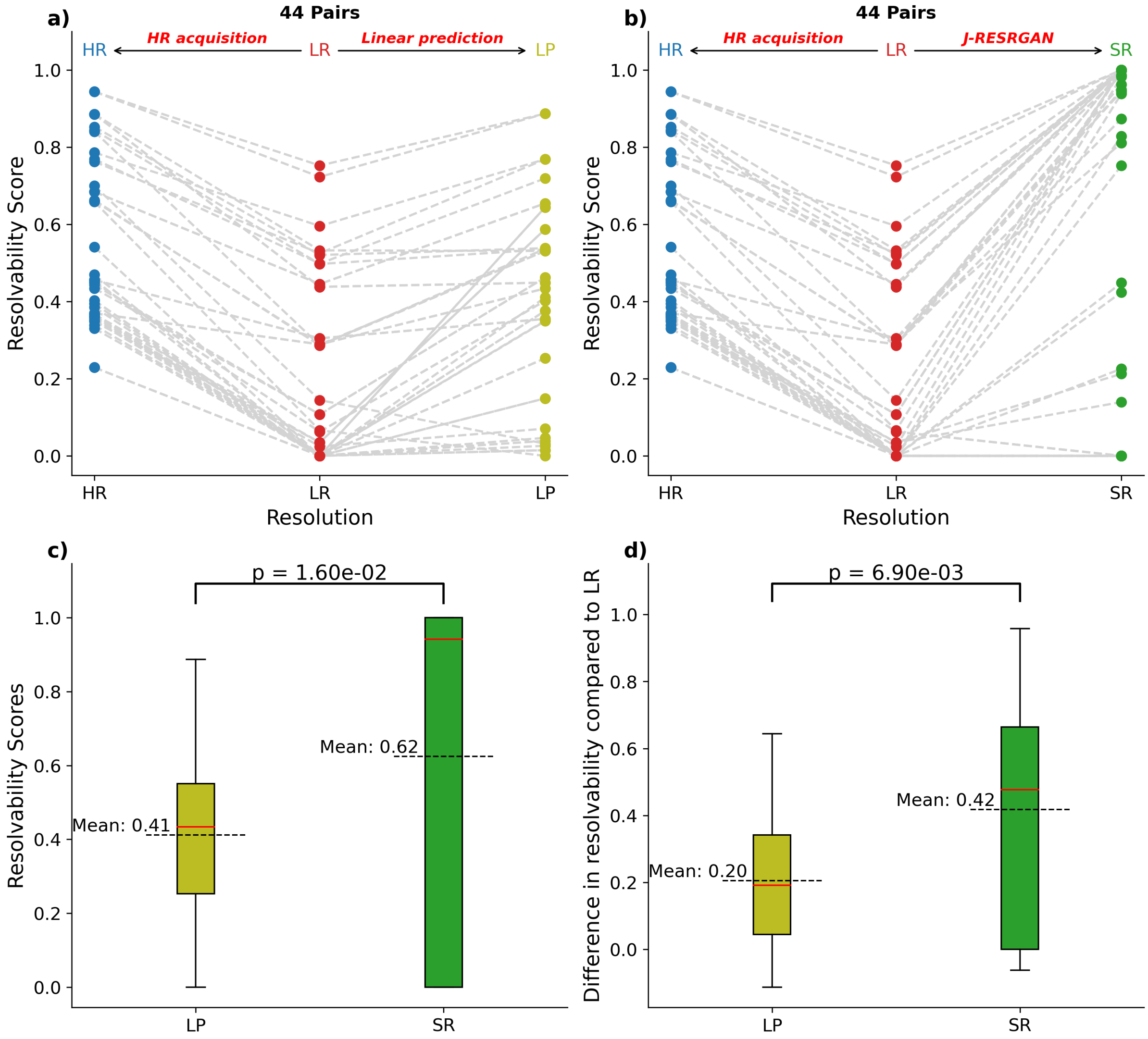

Figure S19. Comparative analysis of resolution enhancement methods: J-RESRGAN and linear prediction, applied to the LR spectrum acquired from a plasma sample with 2 scans (**NS = 2**). The spectra processed by J-RESRGAN and linear prediction are denoted as SR and LP, respectively. All spectra, LR, HR, SR, and LP, were subject to the same level of smoothing to facilitate peak detection during resolvability score calculation. Plot a) illustrates resolvability scores for 44 peak pairs across HR, LR and LP spectra. Plot b) displays resolvability scores for the same peak pairs in HR, LR, and SR spectra. Plot c) presents box plots comparing the resolvability scores obtained by LP and SR, with the SR showing higher median resolvability. Plot d) shows box plots of the difference of resolvability scores relative to LR for both LP and SR, with SR providing a more pronounced improvement in peak separation as indicated by higher median and mean scores. Statistical significance is calculated via a Mann Whitney test, with p = 1.60e-02 for LP versus SR comparison and p = 6.90e-03 for the score difference comparison.

2.12 Comparison between Models with & without Symmetric Loss Function

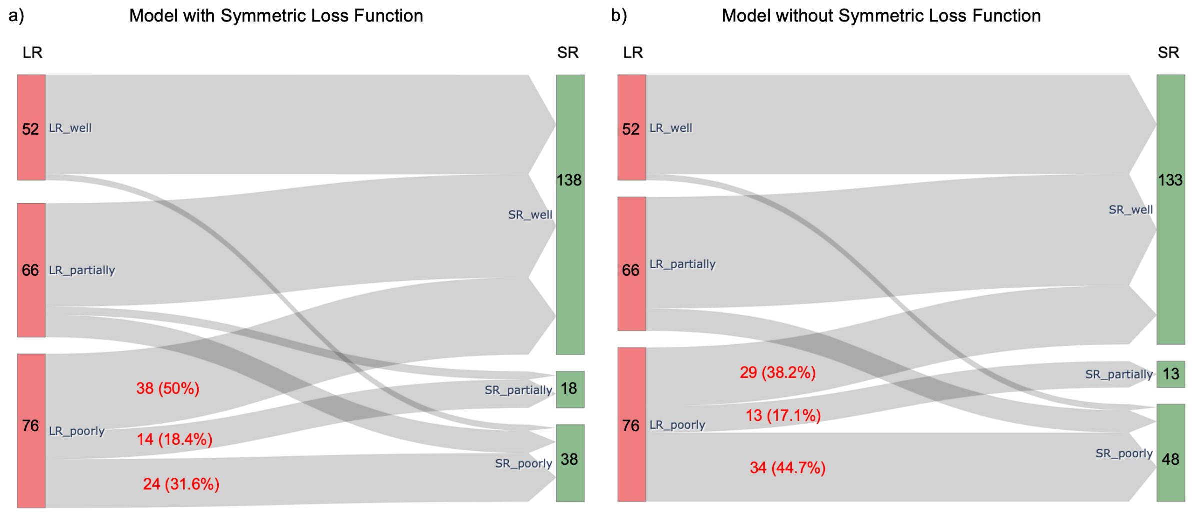

Figure S20. Comparative analysis of peak pair resolvability enhancement by models with and without symmetric loss function. Panel a) illustrates the transformation of peak pairs from LR to SR using the model with a symmetric loss function, whereas panel b) depicts the same without the symmetric loss function. Resolvability scores are categorized into three groups: poorly resolved (0-0.2), partially resolved (0.2-0.6), and well resolved (0.6-1.0). The charts highlight that the model incorporating a symmetric loss function produces a higher proportion of well-resolved peak pairs. For example, in LR, 50% of poorly resolved peak pairs are upgraded to well-resolved in SR when using the symmetric loss function, as compared to 38.2% enhancement achieved by the model lacking this function.

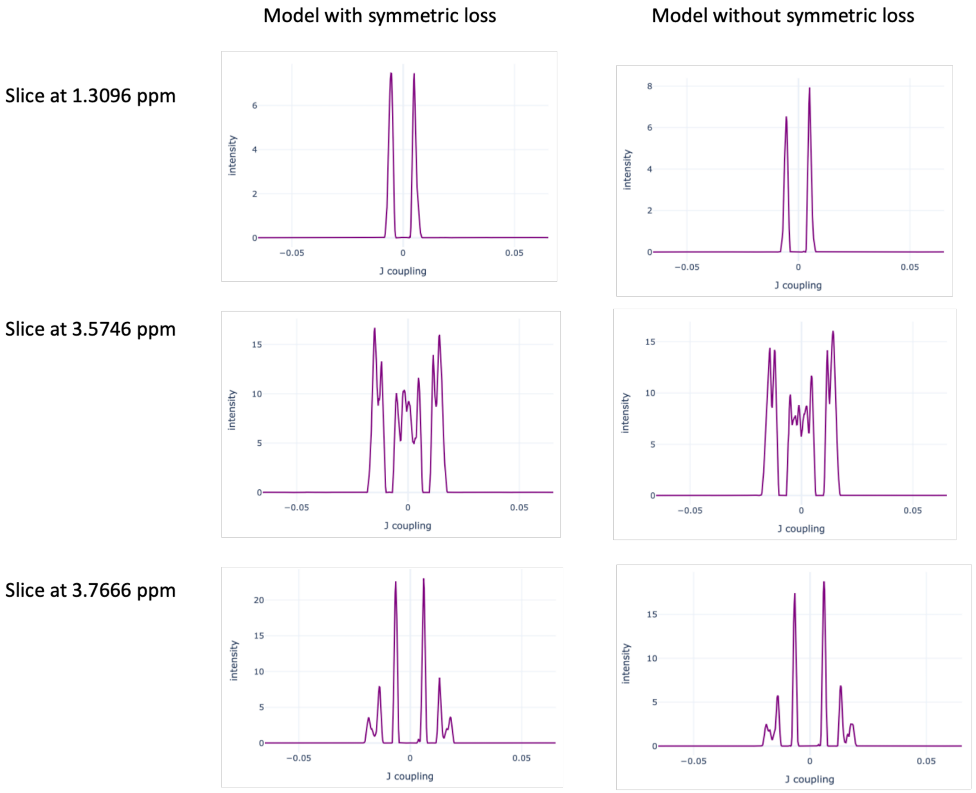

Figure S21. Examples of peak clusters to illustrate the differences between the model with the symmetric loss function and the model without the symmetric loss function.

Reference

[1] Dona, A. C., Jiménez, B., Schäfer, H., Humpfer, E., Spraul, M., Lewis, M. R., ... & Nicholson, J. K. (2014). Precision high-throughput proton NMR spectroscopy of human urine, serum, and plasma for large-scale metabolic phenotyping. *Analytical chemistry*, *86*(19), 9887-9894.

[2] Morris, G. A. (2017). NMR data processing. *Encyclopedia of Spectroscopy and Spectrometry*, 125-133.
